# Mucinous cystic neoplasms and simple mucinous cysts are two distinct precursors of pancreatic cancer: clinicopathological, genomic, and transcriptomic characterization

**DOI:** 10.1002/path.6437

**Published:** 2025-05-15

**Authors:** Antonio Pea, Michele Bevere, Anastasios Gkountakos, Davide Pasini, Denise Fiorini, Andrea Mafficini, Stela Golovco, Michele Simbolo, Serena Pedron, Concetta Sciammarella, Paola Mattiolo, Aldo Mombello, Manuela Villanova, Carlotta Franzina, Francesca Masetto, Calogero Ciulla, Nicola Sperandio, Kohei Fujikura, Masha S. Ahadi, Jaswinder S. Samra, Amber L. Johns, Joanne Verheij, Martijn W.J. Stommel, Hjalmar van Santvoort, Leonor Schubert Santana, Giuseppe Malleo, Michele Milella, Lodewijk A. A. Brosens, Laura D. Wood, David K. Chang, Riccardo De Robertis, Mirko D'Onofrio, Anthony J. Gill, Roberto Salvia, Vincenzo Corbo, Rita T. Lawlor, Aldo Scarpa, Claudio Luchini

**Affiliations:** ^1^ Department of General and Pancreatic Surgery–The Pancreas Institute Verona University Hospital Trust Verona Italy; ^2^ ARC‐Net Research Center University of Verona Verona Italy; ^3^ Department of Engineering for Innovation Medicine University of Verona Verona Italy; ^4^ Department of Medicine University of Verona Verona Italy; ^5^ Department of Diagnostics and Public Health, Section of Pathology University of Verona Verona Italy; ^6^ Department of Pathology, Sol Goldman Pancreatic Cancer Research Center Johns Hopkins School of Medicine Baltimore MD USA; ^7^ Royal North Shore Hospital St Leonards NSW Australia; ^8^ Faculty of Medicine and Health University of Sydney Sydney NSW Australia; ^9^ Cancer Diagnosis and Pathology Group, Kolling Institute of Medical Research, and Department of Anatomical Pathology, NSW Health Pathology Royal North Shore Hospital St Leonards NSW Australia; ^10^ The Garvan Institute of Medical Research and The Kinghorn Cancer Centre Darlinghurst NSW Australia; ^11^ Department of Pathology, Amsterdam UMC University of Amsterdam Amsterdam The Netherlands; ^12^ Department of Surgery Radboud University Medical Center Nijmegen The Netherlands; ^13^ Department of Surgery, Regional Academic Cancer Center Utrecht UMC Utrecht and St Antonius Hospital Utrecht Netherlands; ^14^ Wolfson Wohl Cancer Research Centre, Research Institute of Cancer Sciences University of Glasgow Glasgow UK; ^15^ Department of Pathology, UMC Utrecht, Utrecht University, Utrecht, and Department of Pathology Radboud University Medical Center Nijmegen The Netherlands; ^16^ Sidney Kimmel Comprehensive Cancer Center Johns Hopkins School of Medicine Baltimore MD USA; ^17^ West of Scotland Pancreatic Unit Glasgow Royal Infirmary Glasgow UK; ^18^ Department of Diagnostics and Public Health, Section of Radiology University of Verona Verona Italy

**Keywords:** Mucinous cystic neoplasm, simple mucinous cyst, *CDKN2A*, *RNF43*, MCN, transcriptome, pancreatic precursors, PDAC

## Abstract

Mucinous cystic neoplasms (MCNs) of the pancreas are macroscopic precursors of pancreatic cancer. A similar cystic lesion but lacking the ovarian‐type subepithelial stroma has been recently defined as a simple mucinous cyst (SMC); however, its nature remains unclear. This study aims to define the clinicopathological and molecular profiles of a cohort of MCNs and SMCs of the pancreas and their associated invasive carcinoma. Overall, 23 cases were identified, comprising 19 MCNs and 4 SMCs with co‐occurring invasive carcinoma. A multiregional (two samples from each cystic lesion and one from the adenocarcinoma) DNA and RNA sequencing approach was used. The key findings can be summarized as follows: (1) Molecular association: In 22/23 cases (95.7%), the concomitant mucinous cyst and invasive carcinoma shared specific genomic alterations, establishing for the first time that SMC is a true precursor of pancreatic cancer. (2) Clinical behavior: carcinomas arising from SMC appeared to be more aggressive than those arising from MCN. (3) Mutational profile: both cyst types showed significant similarities to conventional pancreatic ductal adenocarcinoma (PDAC), with *KRAS* and *TP53* the most commonly altered genes. (4) Intracystic heterogeneity: while most molecular alterations were present in both analyzed cystic areas, *RNF43* showed the highest heterogeneity. (5) *CDKN2A*: its alterations were predominantly restricted to the invasive component, suggesting a role in driving the invasion in a subset of cases. *CNKN2A* may also serve as a potential biomarker for identifying high‐risk cysts. (6) RNAseq: most cases showed a switch from the classical to the basal transcriptome subtype during the progression from cystic neoplasms to invasive cancers. These findings establish SMCs as new precursors of pancreatic cancer and provide critical insights into the tumorigenesis of MCNs, with potential immediate implications for tumor taxonomy and clinical management. © 2025 The Author(s). *The Journal of Pathology* published by John Wiley & Sons Ltd on behalf of The Pathological Society of Great Britain and Ireland.

## Introduction

Pancreatic cancer is a deadly malignancy with increasing incidence [1, 2, 3]. Like other epithelial cancers, it arises from noninvasive precursors that can be cured if detected early and appropriately treated [4, 5]. Among the precancers, cystic lesions such as intraductal neoplasms and mucinous cystic neoplasms (MCNs) play a significant role [3]. Intraductal neoplasms encompass intraductal papillary mucinous neoplasms (IPMNs), intraductal oncocytic papillary neoplasms (IOPNs), and intraductal tubulopapillary neoplasms (ITPNs) [3, 6, 7, 8, 9, 10]. All of them comprise neoplastic papillae and communicate with the pancreatic ductal tree [3, 6, 7, 8, 9, 10].

In contrast, MCNs do not harbor connections with the pancreatic ducts [3]. Histologically, they lack papillary projections and have a distinctive ovarian‐type subepithelial stroma [3, 10, 11, 12]. Another subtype of mucinous cysts was identified in the early 2000s [13]. Its definition was recently refined, introducing the terminology of simple mucinous cyst (SMC) [14, 15, 16]. This definition is applied to >1 cm mucinous cysts that do not communicate with the ductal tree and do not have characteristic features of IPMNs or MCNs [15]. While histologically similar to MCNs in terms of cystic appearance and mucinous cystic lining, SMC lacks the subepithelial ovarian‐type stroma. SMCs are hypothesized to be potential cancer precursors, although no cases of SMCs progressing to invasive cancer have been documented [14, 15, 16].

Compared to intraductal lesions, MCNs exhibit less aggressive biological behavior [3, 10, 11, 12, 17, 18, 19, 20], although they can develop into invasive carcinoma in up to 35% of cases [17, 18, 19, 20]. Deciphering the molecular events that drive the malignant transformation of mucinous cysts is crucial for understanding pancreatic oncogenesis and improving cancer prevention strategies. Thus far, in addition to *KRAS* mutations, other genetic drivers have been described, including *PIK3CA* and *RNF43* variations [21, 22, 23, 24]. However, the full spectrum of histological and molecular changes involved in this progression has not been fully elucidated.

In this study we present the largest cohort of molecularly analyzed mucinous cysts of the pancreas (both MCNs and SMCs) associated with invasive carcinoma and define the clinicopathological, genomic, and transcriptomic profiles of the lesions. The study findings provide novel insights into pancreatic oncogenesis, with potentially immediate implications for tumor taxonomy, prevention strategies, and therapeutic approaches.

## Materials and Methods

### Ethics approval

This study was approved by the Ethics Committees of therespective institutions/Verona Ethics Committee (2610‐CESC). Patients gave informed consent for the study. All procedures were in accordance with the Helsinki Declaration of 1975, as revised in 1983 (https://www.wma.net/what‐we‐do/medical‐ethics/declaration‐of‐helsinki/doh‐oct1983/ date last accessed 18/04/2025).

### Case selection and clinicopathological analysis

The following electronic databases were searched for cases of pancreatic mucinous cysts associated with invasive carcinoma: Verona University Hospital (Verona, Italy), Radboud University Medical Center (Nijmegen, The Netherlands), Utrecht University Medical Center (Utrecht, The Netherlands), Amsterdam University Medical Center (Amsterdam, The Netherlands), Johns Hopkins Hospital (Baltimore, MD, USA), and Royal North Shore Hospital (Sydney, NSW, Australia).

For the histological definition of MCN, we applied the tumors classification criteria of the World Health Organization [3]. For correctly identifying SMCs, we applied the criteria adopted by the study with the largest cohort of this entity [15]. Furthermore, we considered only pancreatic cysts that were entirely sampled and submitted for histological analysis, since the presence of ovarian‐type stroma (an essential feature for the differential diagnosis with MCNs) or of neoplastic papillae (a crucial feature for the differential diagnosis with IPMNs) can be focal. Only cases in which tissue was available for molecular analysis were included. Overall, 23 cases were therefore selected, centrally reviewed, and confirmed by two pancreatic pathologists (AS, CL). Clinical data, including prognostic indices, were obtained from medical records and electronic databases.

### Tissue selection and preparation for molecular analyses

To better understand tumor heterogeneity and evolution, a multiregional sequencing approach was adopted. Therefore, for each case we selected an area of the cyst not adjacent to the invasive tumor and with low‐grade dysplasia (LGD; this area was called C1), another area of the cyst close to, but not overlapping with, the invasive tumor and with high‐grade dysplasia (HGD; called C2), and an area with only the invasive tumor (called AC) for molecular analysis. DNA and RNA were prepared from formalin‐fixed paraffin‐embedded (FFPE) neoplastic tissues enriched via microscope‐guided manual microdissection.

### Multiregional DNA sequencing

DNA next‐generation sequencing (NGS) was performed on all selected regions (C1, C2, and AC) using the previously described SureSelectXT HS CD Glasgow Cancer Core assay (www.agilent.com), hereafter referred to as CORE [25, 26, 27], which spans 1.8 megabases of the genome, targeting 174 genes for somatic mutations, copy number alterations, and structural rearrangements. Details of the target genes are presented in the supplementary material, Table S1 and Supplementary materials and methods. Sequencing was performed using NextSeq 500 (Illumina, San Diego, CA, USA).

### Multiregional RNA sequencing

RNA sequencing (RNAseq) was performed using established methods (see also Supplementary materials and methods) [28]. In brief, reads were aligned to the GRCh38 genome using STAR v2.7 [29]. RSEM transcript quantification was imported in R. Subtyping was based on the highest ssGSEA score between the basal and classical gene programs of pancreatic ductal adenocarcinoma (PDAC).

### NGS, fluorescent *in situ* hybridization (FISH), and immunohistochemistry (IHC)

In patients with *CDKN2A*/*B* and *BRCA1*/*2* mutations, NGS was also performed on healthy tissues to verify the presence of germline alterations.

Specific FISH analyses were performed for CDKN2A as previously described [30] (see also Supplementary materials and methods).

For *ERBB2* amplification, specific immunohistochemical analysis of HER2 (HercepTest, Dako, Jena, Germany) was performed for further validation and scored as previously described [31].

All SMCs were analyzed with IHC for estrogen receptor (clone: 6F11; dilution: prediluted; source: Leica, Wetzlar, Germany), progesterone receptor (16, prediluted, Leica), and alpha‐inhibin (R1; prediluted; Leica) to verify the possible presence of regressed ovarian‐type stroma.

Additional IHC was performed on all cysts for investigating the expression of mucins MUC1, MUC2, MUC4. MUC5AC, and MUC6, as previously described [32] (see also Supplementary materials and methods).

### Comparative and survival analysis

An additional cohort composed of MCNs with LGD and with HGD but without an associated invasive carcinoma was collected to compare their clinicopathological features with those of MCNs with an associated invasive carcinoma (Student's *t*‐test). Survival analysis was based on univariate Cox regression analyses and on a multivariate model (see Supplementary materials and methods).

## Results

### Clinicopathologic analysis

**Figure 1:**
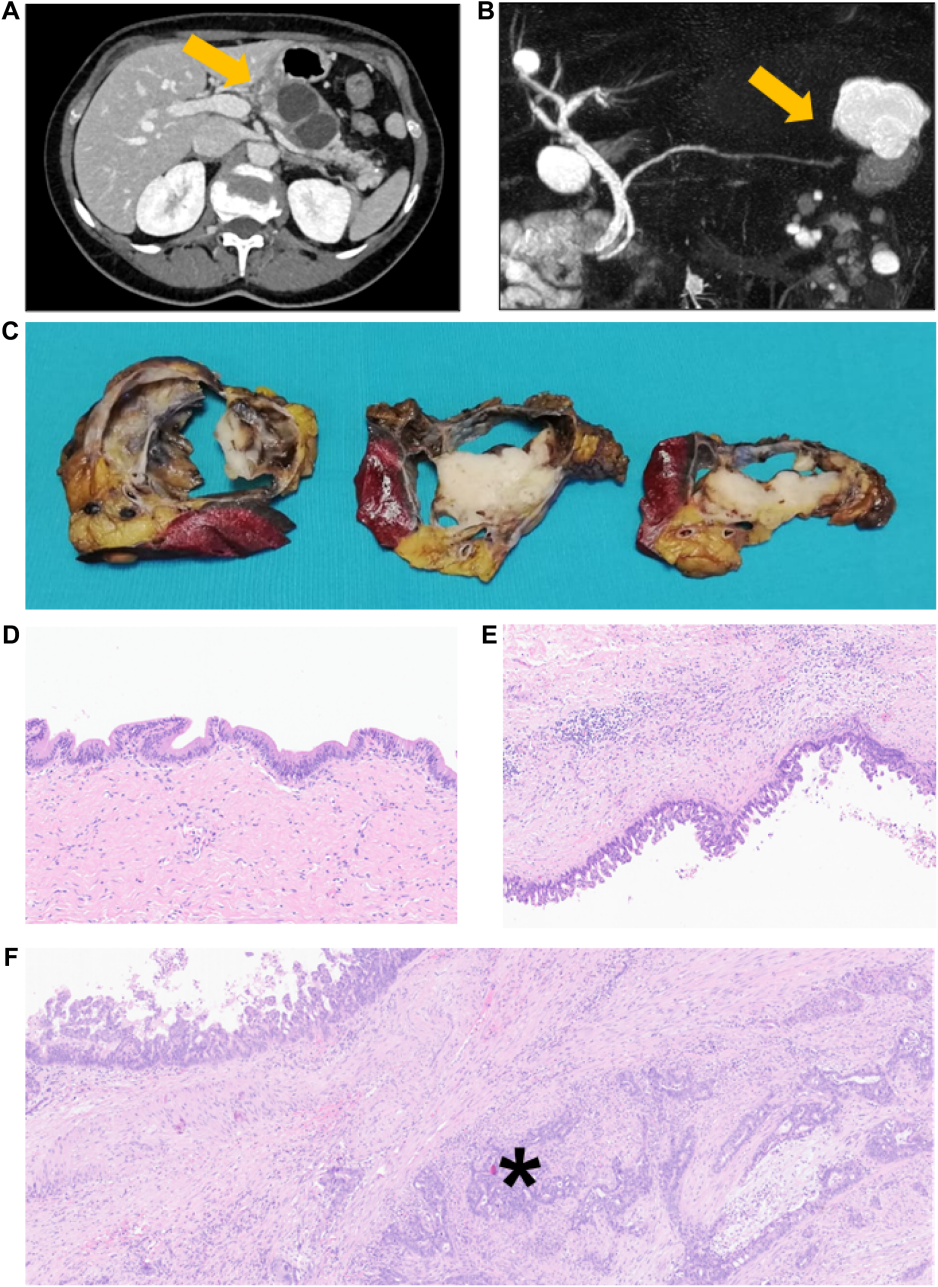
Macroscopic and microscopic features of simple mucinous cyst of the pancreas with associated invasive carcinoma. (A) CT scan showing a biloculated cyst in the pancreatic body (patient no. 21). (B) Magnetic resonance cholangiopancreatography revealed a multiloculated cyst in the tail of the pancreas, with no evidence of connection to the pancreatic ducts (patient no. 22). (C) Macroscopic features of a simple mucinous cyst located in the tail of the pancreas (patient no. 22). A multilocular cyst is visible, with the whitish and firm tissue representing the invasive carcinoma. A small fragment of spleen is also included in the image. (D–F) Histological features of simple mucinous cyst of the pancreas. Notably, the characteristic ovarian‐type stroma, typical of mucinous cystic neoplasms, is absent (hematoxylin–eosin staining). (D) Area showing low‐grade dysplasia (original magnification, 10×); (E) Another area, distant from the area in panel (D), demonstrating high‐grade dysplasia (10×); (F). Simple mucinous cyst with associated invasive carcinoma, indicated by an asterisk (10×).

A total of 23 cases of pancreatic mucinous cysts (both MCNs and SMCs) with co‐occurring invasive carcinomas were included in the current study. The cohort consisted of 18 MCNs with invasive carcinoma (cases no. 1–18), four SMCs with invasive carcinoma (cases no. 19–22), and one case with co‐occurring MCN, IPMN, and invasive carcinoma (case no. 23). Molecular characterization of the last case (no. 23) clarified the association between IPMN and carcinoma (see below); therefore, the mean values and numerical summary of the current case series were calculated excluding this case. Histopathological and radiological features of MCNs and SMCs with associated invasive carcinoma are shown in the supplementary material Figures S1–S4, and Figure 1.

The clinicopathological data are summarized in Table 1. Statistical comparisons between the MCN and the SMC cohorts (chi‐square test) revealed significant differences between the two groups in the prevalence of tumor stage I (*p* = 0.02), female sex (*p* = 0.02), and nodal metastases (*p* = 0.01), with stage I and female sex more common in the MCN group and nodal metastasis more prevalent in the SMC group.

**Table 1:** Clinicopathological data of all cases of the current series

ID case	Sex, age	Site in the pancreas	Histology CL	Histology of cancer and grading	Size CL (size IC)	pTNM	Tumor stage	VI	PNI	R	TTR (months) and site	SS/CSS (months)
1	F, 46	B‐T	MCN	Tubular, G1	9.0 (0.5)	pT1aN0M0	IA	No	No	R0	13, SP	DOD (45)
2	F, 64	T	MCN	Tubular, G2	9.0 (NA)	pTxNxM0	NA	Yes	Yes	NA	NA	DOD (9)
3	F, 63	B‐T	MCN	Tubular, G2	10.0 (0.6)	pT1bN0M0	IA	No	No	R0	No relapse	AF (166)
4	F, 58	T	MCN	Tubular, G3	11.5 (0.6)	pT1bN0M0	IA	No	No	R0	NA	NA
5	F, 58	B‐T	MCN	Anaplastic, G4	15.0 (0.7)	pT1bN0M0	IA	No	No	R0	No relapse	AF (31)
6	F, 64	T	MCN	UCOGC, G4	4.0 (2.2)	pT2N0M0	IB	Yes	No	R0	No relapse	AF (104)
7	F, 45	B	MCN	Tubular, G2	6.0 (0.9)	pT1bN0M0	IA	No	No	R0	No relapse	AF (31)
8	F, 54	T	MCN	Tubular, G2	5.0 (1.3)	pT1cN0M0	IA	No	No	R0	No relapse	AF (154)
9	F, 63	T	MCN	Tubular, G2	4.5 (2.5)	pT2N0M0	IB	Yes	Yes	R0	No relapse	AF (145)
10	F, 61	B‐T	MCN	UCOGC, G4	6.0 (2.8)	pT2N0M0	IB	Yes	No	R1 (RP)	39, LivM	DOD (51)
11	F, 74	T	MCN	Tubular, G1	5.5 (0.5)	pT1aN0M0	IA	No	Yes	R0	No relapse	AF (72)
12	M, 74	T	MCN	Tubular, G2	11.5 (4.4)	pT3N2M0	III	Yes	Yes	R0	NA	DOD (9)
13	F, 44	T	MCN	Tubular, G1	8.0 (0.5)	pT1aN0M0	IA	No	No	R0	No relapse	AF (156)
14	F, 57	H	MCN	Tubular, G2	7.0 (4.2)	pT3N1M0	IIB	Yes	Yes	R0	NA	DOD (36)
15	F, 61	B‐T	MCN	Tubular, G2	3.5 (2.2)	pT2N1M0	IIB	Yes	Yes	R0	18, RP, LAD	AD (18)
16	F, 53	T	MCN	Tubular, G2	4.5 (0.8)	pT1bN0M0	IA	Yes	Yes	R0	No relapse	AF (46)
17	M, 65	B‐T	MCN	Tubular, G2	8.0 (3.5)	pT2N0M1 (LadM)	IV	Yes	Yes	R0	8, P	AD (8)
18	F, 63	T	MCN	UCOGC, G4	9.0 (4.1)	pT3N1M0	IIB	Yes	Yes	R0	No relapse	AF (40)
19	M, 86	B‐T	SMC	Tubular, G2	3.7 (2.8)	pT2N1M0	IIB	Yes	Yes	R0	16, LivM	DOD (19)
20	M, 79	H	SMC	Tubular, G2	4.0 (2.5)	pT2N2M0	III	Yes	Yes	R0	NA	DOD (33)
21	F, 57	B	SMC	Tubular, G2	3.7 (2.2)	pT2N1M0	IIB	Yes	Yes	R0	39, LungM	AD (48)
22	M, 66	T	SMC	MP, G3	4.5 (3.3)	pT2N1M0	IIB	Yes	Yes	R0	27, LungM	AD (27)
23	F, 57	T	MCN	Tubular, G1	2.6	pT1cN0M0	IA	Yes	Yes	R0	No relapse	AF (60)
		B	IPMN		3.0 (1.3)							

Abbreviations: AD, alive with disease; AF, alive free of disease; DOD, dead of disease; LadM, distant metastasis at the left adrenal gland; LivM, liver metastasis; LungM, lung metastasis; MP, micropapillary; NA, not available/applicable; P, peritoneal; RP, retroperitoneal; Size CL, size of the cystic lesion (cm); size IC, size of the invasive carcinoma (cm); SP, systemic progression; SS/CCS, survival status/cancer‐specific survival; TTR, time to relapse; UCOGC, undifferentiated carcinoma with osteoclast‐like giant cells.

### Multiregional DNA sequencing

**Figure 2:**
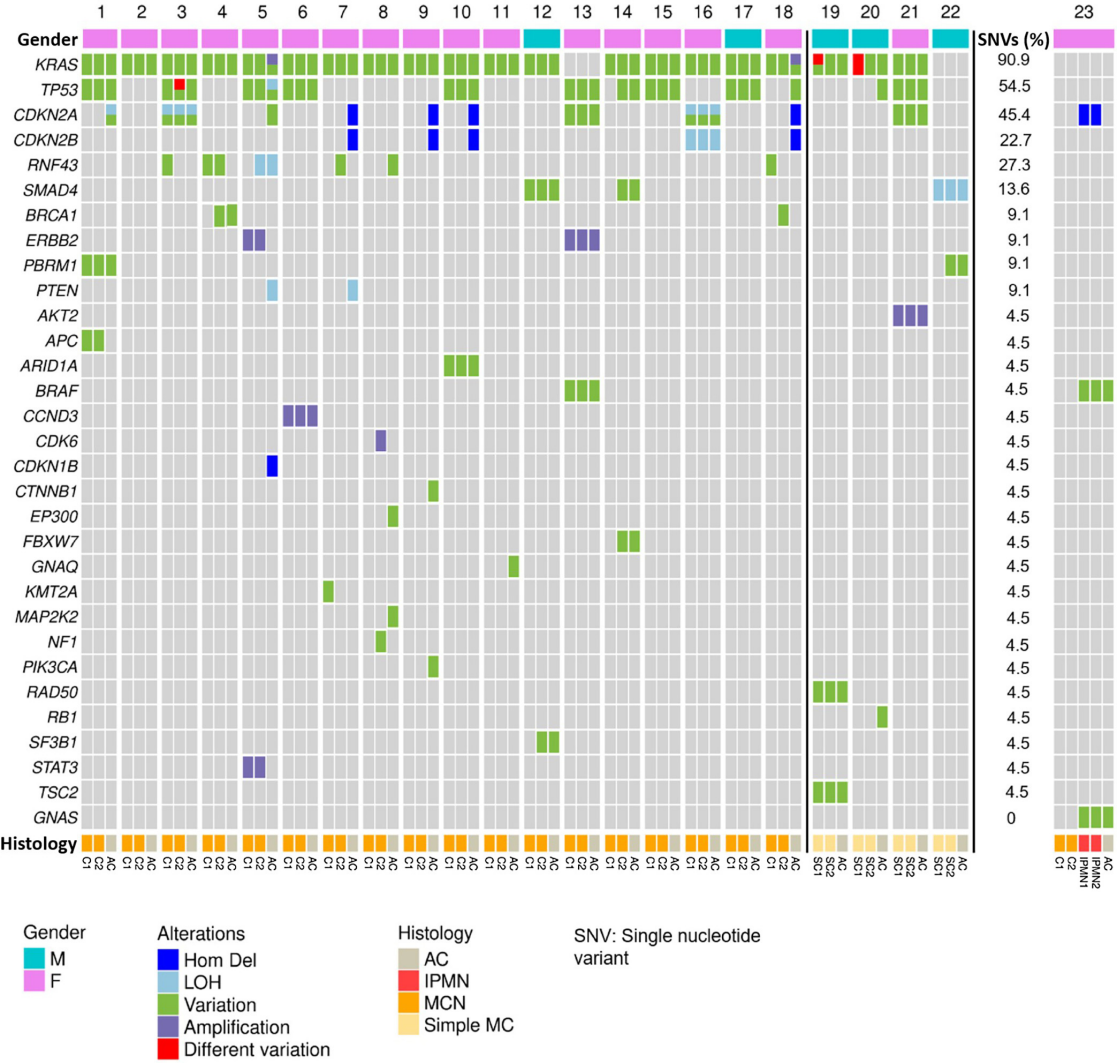
Oncoprint of the genomic alterations detected by next‐generation sequencing in all cases. AC, associated carcinoma; F, female; Hom Del, homozygous deletion; IPMN, intraductal papillary mucinous neoplasm; LOH, loss of heterozygosity; M, male; MCN, mucinous cystic neoplasm; Simple MC, simple mucinous cyst.

The molecular findings based on multiregional DNA NGS are summarized in supplementary material, Table S2 and Table 2, and graphically represented in Figure 2 (71 analyzed samples, including two samples from the IPMN of case no. 23). In 22 cases (cases no. 1–22) we observed that co‐occurring mucinous cysts and invasive adenocarcinomas shared specific molecular alterations, demonstrating that the lesions were molecularly associated. In the remaining case (case no. 23) harboring co‐occurring MCN, IPMN, and invasive cancer, NGS showed a molecular association between IPMN and invasive cancer, since they share the same molecular alterations involving *GNAS* and *BRAF*. Consequently, this case was excluded from further analysis.

**Table 2:** Pathogenic/likely pathogenic mutations and gene copy‐number variations identified in pancreatic cysts and associated invasive carcinoma

ID case	Histology and area	Clinically relevant SNV					CNV			
		Gene	Variation	Mutation type	Freq (%)	Class	Gene	Variation	# of copies	Class
1	MCN‐C1	*KRAS*	p.G12R	Substitution–missense	35	5	None			
		*TP53*	p.R248Q	Substitution–missense	57	5				
		*APC*	p.T1556fs*3	Insertion–frameshift	55	5				
		*PBRM1*	p.G1430D	Substitution–missense	42	4				
	MCN‐C2	*KRAS*	p.G12R	Substitution–missense	13	5	None			
		*TP53*	p.R248Q	Substitution–missense	10	5				
		*APC*	p.T1556fs*3	Insertion–frameshift	7	5				
		*PBRM1*	p.G1430D	Substitution–missense	49	4				
	AC	*KRAS*	p.G12R	Substitution–missense	24	5	None			
		*CDKN2A*	p.W110*	Substitution–stop‐gain	3	5				
		*TP53*	p.R248Q	Substitution–missense	23	5				
		*PBRM1*	p.G1430D	Substitution–missense	50	4				
2	MCN‐C1	*KRAS*	p.G12D	Substitution–missense	45	5	None			
	MCN‐C2	*KRAS*	p.G12D	Substitution–missense	55	5	None			
	AC	*KRAS*	p.G12D	Substitution–missense	23	5	None			
3	MCN‐C1	*KRAS*	p.G12V	Substitution–missense	31	5	*CCND3*	Gain	4	4
		*CDKN2A*	p.L78fs*41	Deletion–frameshift	40	5				
		*TP53*	p.K132N	Substitution–missense	6	5				
		*RNF43*	p.S468*	Substitution–stop‐gain	10	4				
	MCN‐C2	*KRAS*	p.G12V	Substitution–missense	30	5	*CCND3*	Gain	4	4
		*CDKN2A*	p.L78fs*41	Deletion–frameshift	25	5				
		*TP53*	p.Y220C	Substitution–missense	12	5				
		*TP53*	p.E285K	Substitution–missense	10	5				
	AC	*KRAS*	p.G12V	Substitution–missense	41	5	None			
		*CDKN2A*	p.L78fs*41	Deletion–frameshift	48	5				
		*TP53*	p.V157G	Substitution–missense	36	5				
4	MCN‐C1	*KRAS*	p.G12R	Substitution–missense	24	5	None			
		*RNF43*	c.849 + 1G > C	Substitution–splice site	4	4				
	MCN‐C2	*KRAS*	p.G12R	Substitution–missense	30	5	None			
		*RNF43*	p.L21fs*30	Substitution–splice site	27	5				
		*BRCA1*	p.I1159fs*50	Deletion–frameshift	8	5				
	AC	*KRAS*	p.G12R	Substitution–missense	20	5	None			
		*BRCA1*	p.I1159fs*50	Deletion–frameshift	7	5				
5	MCN‐C1	*KRAS*	p.G12D	Substitution–missense	13	5	*ERBB2*	Amp	8	5
		*TP53*	p.R273C	Substitution–missense	17	5	*STAT3*	Amp	6	3
	MCN‐C2	*KRAS*	p.G12D	Substitution–missense	24	5	*ERBB2*	Amp	10	5
		*TP53*	p.R273C	Substitution–missense	29	5	*STAT3*	Amp	8	3
							*RNF43*	LOH	1	4
	AC	*KRAS*	p.G12D	Substitution–missense	75	5	*KRAS*	Amp	6	5
		*CDKN2A*	p.H83Y	Substitution–missense	64	5	*CDKN1B*	Hom Del	0	5
		*TP53*	p.R273C	Substitution–missense	41	5	*TP53*	LOH	1	4
							*PTEN*	LOH	1	4
							*RNF43*	LOH	1	4
6	MCN‐C1	*KRAS*	p.G12V	Substitution–missense	27	5	*CCND3*	Amp	10	5
		*TP53*	c.673‐1G>C	Substitution–splice site	12	5				
	MCN‐C2	*KRAS*	p.G12V	Substitution–missense	25	5	*CCND3*	Amp	10	5
		*TP53*	c.673‐1G>C	Substitution–splice site	10	5				
	AC	*KRAS*	p.G12V	Substitution–missense	33	5	*CCND3*	Amp	10	5
		*TP53*	c.673‐1G>C	Substitution–splice site	43	5				
7	MCN‐C1	*KRAS*	p.G12D	Substitution–missense	24	5	*RNF43*	LOH	1	4
		*KMT2A*	p.H2774fs*13	Deletion–frameshift	34	4				
		*ATM*	p.C74fs*2	Deletion–frameshift	8	4				
	MCN‐C2	*KRAS*	p.G12D	Substitution–missense	33	5	None			
		*RNF43*	p.Q84*	Substitution–stop‐gain	15	4				
	AC	*KRAS*	p.G12D	Substitution–missense	35	5	*CDKN2A*	Hom Del	0	5
							*CDKN2B*	Hom Del	0	5
							*KRAS*	Gain	3	4
							*IGF1R*	Gain	3	4
							*PTEN*	LOH	1	4
8	MCN‐C1	*KRAS*	p.G12V	Substitution–missense	61	5	*KRAS*	Gain	4	4
	MCN‐C2	*KRAS*	p.G12V	Substitution–missense	32	5	*CDK6*	Amp	50	5
		*NF1*	p.E2174fs*46	Deletion–frameshift	8	5				
	AC	*KRAS*	p.G12V	Substitution–missense	46	5	None			
		*RNF43*	p.R113*	Substitution–stop‐gain	31	5				
		*MAP2K2*	p.Y134C	Substitution–missense	10	5				
		*EP300*	p.D1399N	Substitution–missense	7	4				
9	MCN‐C1	*KRAS*	p.G12D	Substitution–missense	16	5	None			
	MCN‐C2	*KRAS*	p.G12D	Substitution–missense	16	5	None			
	AC	*KRAS*	p.G12D	Substitution–missense	60	5	*CDKN2A*	Hom Del	0	5
		*CTNNB1*	p.S45F	Substitution–missense	37	5	*CDKN2B*	Hom Del	0	5
		*PIK3CA*	p.E545K	Substitution–missense	40	5				
		*PIK3CA*	p.E726K	Substitution–missense	31	5				
10	MCN‐C1	*KRAS*	p.G12D	Substitution–missense	29	5	None			
		*TP53*	c.375 + 5G>T	Substitution–splice site	41	5				
		*ARID1A*	p.G804Vfs*30	Insertion–frameshift	15	4				
	MCN‐C2	*KRAS*	p.G12D	Substitution–missense	14	5	None			
		*TP53*	c.375 + 5G>T	Substitution–splice site	17	5				
		*ARID1A*	p.G804Vfs*30	Insertion–frameshift	7	4				
	AC	*KRAS*	p.G12D	Substitution–missense	49	5	*CDKN2A*	Hom Del	0	5
		*TP53*	c.375 + 5G>T	Substitution–splice site	78	5	*CDKN2B*	Hom Del	0	5
		*ARID1A*	p.G804Vfs*30	Insertion–frameshift	33	4				
11	MCN‐C1	*KRAS*	p.Q61H	Substitution–missense	17	5	None			
	MCN‐C2	*KRAS*	p.Q61H	Substitution–missense	15	5	None			
	AC	*KRAS*	p.Q61H	Substitution–missense	4	5	None			
		*GNAQ*	p.R183Q	Substitution–missense	3	5				
12	MCN‐C1	*KRAS*	p.G12V	Substitution–missense	5	5	None			
		*SMAD4*	p.L536fs*4	Deletion–frameshift	5	4				
	MCN‐C2	*KRAS*	p.G12V	Substitution–missense	10	5	None			
		*SF3B1*	p.K700E	Substitution–missense	8	5				
		*SMAD4*	p.Y162fs*4	Deletion–frameshift	3	4				
	AC	*KRAS*	p.G12V	Substitution–missense	9	5	None			
		*SF3B1*	p.K700E	Substitution–missense	8	5				
		*SMAD4*	p.Y162fs*4	Deletion–frameshift	10	4				
13	MCN‐C1	*BRAF*	p.L597Q	Substitution–missense	58	5	*ERBB2*	Amp	48	5
		*TP53*	p.V157F	Substitution–missense	68	5				
		*CDKN2A*	p.L130Q	Substitution–missense	34	4				
	MCN‐C2	*BRAF*	p.L597Q	Substitution–missense	28	5	*ERBB2*	Amp	59	5
		*TP53*	p.V157F	Substitution–missense	59	5				
		*CDKN2A*	p.L130Q	Substitution–missense	35	4				
	AC	*BRAF*	p.L597Q	Substitution–missense	25	5	*ERBB2*	Amp	37	5
		*TP53*	p.V157F	Substitution–missense	27	5				
		*CDKN2A*	p.L130Q	Substitution–missense	16	4				
14	MCN‐C1	*KRAS*	p.G12R	Substitution–missense	33	5	None			
	MCN‐C2	*KRAS*	p.G12R	Substitution–missense	21	5	None			
		*TP53*	p.R248W	Substitution–missense	18	5				
		*FBXW7*	p.R465C	Substitution–missense	24	5				
		*SMAD4*	p.Q534_L535del	Deletion–in‐frame	7	4				
	AC	*KRAS*	p.G12R	Substitution–missense	12	5	None			
		*TP53*	p.R248W	Substitution–missense	17	5				
		*FBXW7*	p.R465C	Substitution–missense	13	5				
		*SMAD4*	p.Q534_L535del	Deletion–in‐frame	8	4				
15	MCN‐C1	*KRAS*	p.G12D	Substitution–missense	10	5	None			
		*TP53*	p.D48fs*4	Insertion–frameshift	10	5				
	MCN‐C2	*KRAS*	p.G12D	Substitution–missense	11	5	None			
		*TP53*	p.D48fs*4	Insertion–frameshift	8	5				
	AC	*KRAS*	p.G12D	Substitution–missense	2	5	None			
		*TP53*	p.D48fs*4	Insertion–frameshift	1	5				
16	MCN‐C1	*KRAS*	p.G12R	Substitution–missense	16	5	*CDKN2A*	LOH	1	4
		*CDKN2A*	p.E27*	Substitution–stop‐gain	9	5	*CDKN2B*	LOH	1	4
	MCN‐C2	*KRAS*	p.G12R	Substitution–missense	40	5	*CDKN2A*	LOH	1	4
		*CDKN2A*	p.E27*	Substitution–stop‐gain	23	5	*CDKN2B*	LOH	1	4
	AC	*KRAS*	p.G12R	Substitution–missense	6	5	*CDKN2A*	LOH	1	4
		*CDKN2A*	p.E27*	Substitution–stop‐gain	3	5	*CDKN2B*	LOH	1	4
17	MCN‐C1	*KRAS*	p.G12V	Substitution–missense	32	5	None			
		*TP53*	p.S90fs*59	Insertion–frameshift	32	5				
	MCN‐C2	*KRAS*	p.G12V	Substitution–missense	16	5	None			
		*TP53*	p.S90fs*59	Insertion–frameshift	26	5				
	AC	*KRAS*	p.G12V	Substitution–missense	30	5	None			
		*TP53*	p.S90fs*59	Insertion–frameshift	25	5				
18	MCN‐C1	*KRAS*	p.Q61H	Substitution–missense	16	5	None			
		*RNF43*	p.R371*	Substitution–stop‐gain	13	5				
	MCN‐C2	*KRAS*	p.Q61H	Substitution–missense	10	5	None			
		*BRCA1*	p.I1159fs*50	Deletion–frameshift	4	5				
	AC	*KRAS*	p.Q61H	Substitution–missense	84	5	*MYC*	Gain	4	5
		*TP53*	p.N247I	Substitution–missense	47	5	*CDKN2A*	Hom Del	0	5
							*CDKN2B*	Hom Del	0	5
							*KRAS*	Amp	10	5
19	SMC‐C1	*KRAS*	p.G12D	Substitution–missense	6	5	None			
		*KRAS*	p.G12R	Substitution–missense	4	5				
		*RAD50*	p.N1310Kfs*10	Complex–frameshift	15	4				
		*TSC2*	p.V1298Wfs*27	Deletion–frameshift	5	4				
	SMC‐C2	*KRAS*	p.G12D	Substitution–missense	6	5	None			
		*RAD50*	p.N1310Kfs*10	Complex–frameshift	18	4				
		*TSC2*	p.V1298Wfs*27	Deletion–frameshift	7	4				
	AC	*KRAS*	p.G12D	Substitution–missense	6	5	None			
		*RAD50*	p.N1310Kfs*10	Complex–frameshift	15	4				
		*TSC2*	p.V1298Wfs*27	Deletion–frameshift	6	4				
20	SMC‐C1	*KRAS*	p.G12D	Substitution–missense	12	5	None			
	SMC‐C2	*KRAS*	p.G12V	Substitution–missense	5	5	None			
	AC	*KRAS*	p.G12V	Substitution–missense	8	5	None			
		*TP53*	p.V272M	Substitution–missense	12	5				
		*RB1*	p.R661Q	Substitution–missense	11	4				
21	SMC‐C1	*KRAS*	p.G12R	Substitution–missense	10	5	*AKT2*	Amp	13	5
		*TP53*	c.‐26 + 1G>A	Substitution–splice site	13	4				
		*CDKN2A*	p.D92fs*28	Insertion–frameshift	13	4				
	SMC‐C2	*KRAS*	p.G12R	Substitution–missense	18	5	*AKT2*	Amp	10	5
		*TP53*	c.‐26 + 1G>A	Substitution–splice site	26	4	*CCND1*	Gain	4	
		*CDKN2A*	p.D92fs*28	Insertion–frameshift	23	4	*FGF19*	Gain	4	
	AC	*KRAS*	p.G12R	Substitution–missense	20	5	*AKT2*	Amp	8	5
		*TP53*	c.‐26 + 1G>A	Substitution–splice site	24	4				
		*CDKN2A*	p.D92fs*28	Insertion–frameshift	24	4				
22	SMC‐C1	None					*SMAD4*	LOH	1	4
	SMC‐C2	*PBRM1*	p.E764*	Substitution–stop‐gain	1	4	*SMAD4*	LOH	1	4
	AC	*PBRM1*	p.E764*	Substitution–stop‐gain	26	4	*SMAD4*	LOH	1	4
23	MCN‐C1	None					None			
	MCN‐C2	None					None			
	IPMN‐C1	*GNAS*	p.R201C	Substitution–missense	48	5	CDKN2A	Hom Del	0	5
		*BRAF*	p.V600_K601delinsE	Deletion–in‐frame	32	5				
	IPMN‐C2	*GNAS*	p.R201C	Substitution–missense	65	5	CDKN2A	Hom Del	0	5
		*BRAF*	p.V600_K601delinsE	Deletion–in‐frame	32	5	GNAS	Gain (exon 1)	5	4
	AC	*GNAS*	p.R201C	Substitution–missense	30	5	None			
		*BRAF*	p.V600_K601delinsE	Deletion–in‐frame	30	5				

Abbreviations: 1, benign (not reported); 2, likely benign (not reported); 3, variant of unknown significance (VUS); 4, likely pathogenic; 5, pathogenic; amplification: >5 copies; C1, area of the cyst non‐adjacent to the invasive cancer; C2, area of the cyst adjacent to the invasive cancer; Class, clinical impact class according to ACMG/AMP Guidelines; CNV, Copy Number Variations; gain, >2 copies; Hom Del, 0 copies; LOH, 1 copy; MCN, mucinous cystic neoplasm; SMC, simple mucinous cyst; SNV, Small Nucleotide Variants.

The molecular landscape of mucinous cysts and their associated invasive cancers is dominated by recurrent alterations in different key genes. *KRAS* mutations were identified in 90.9% of the cases (20/22), including 17/18 MCNs and 3/4 SMCs. *TP53* mutations were present in 54.6% of the cases (12/22), including 10/18 MCNs and 2/4 SMCs. *CDKN2A* was altered in 45.5% of the cases (10/22), predominantly in the invasive components (9/18 MCNs and 1/4 SMCs). *RNF43* and *CDKN2B* alterations were observed only in MCNs (6/18 cases and 5/18 cases, respectively). No statistically significant differences were observed in the prevalence of genetic alterations between MCNs and SMCs (chi‐square test).

In terms of intracystic/intratumor heterogeneity, 71.2% (37/52) of the molecular alterations detected were present in both analyzed cystic areas. *RNF43* was altered in MCNs only and exhibited the highest rate of heterogeneity, with alterations present in only one of the two cystic regions in 80% of the cases (4/5). This situation was significantly different from one observed in MCNs for the two most commonly altered genes, namely *KRAS* and *TP53*, which shared the same mutations in both the C1‐C2 cystic areas in all cases for *KRAS* (17/17) or in almost all cases for *TP53* (8/9) (*p* < 0.05, chi‐squared test). In two individual cases, the two cystic regions shared the same *KRAS* or *TP53* mutation, but one of the areas also harbored an additional mutation in the same gene. When comparing the genetic landscape of mucinous cysts to that of their associated invasive carcinomas, 30.1% (22/73) of genetic alterations were exclusive to the invasive component. *CDKN2A* was the most frequently altered gene restricted to the invasive component (six cases), followed by *CDKN2B* (four cases) and *TP53* (two cases) (supplementary material, Figure S5).

### Multiregional RNA sequencing

Overall, 11 cases (33 samples: three regions per case, two areas of the cyst and one of the invasive carcinomas) were available for RNAseq. Of these, seven were MCNs and four were SMCs.

**Figure 3:**
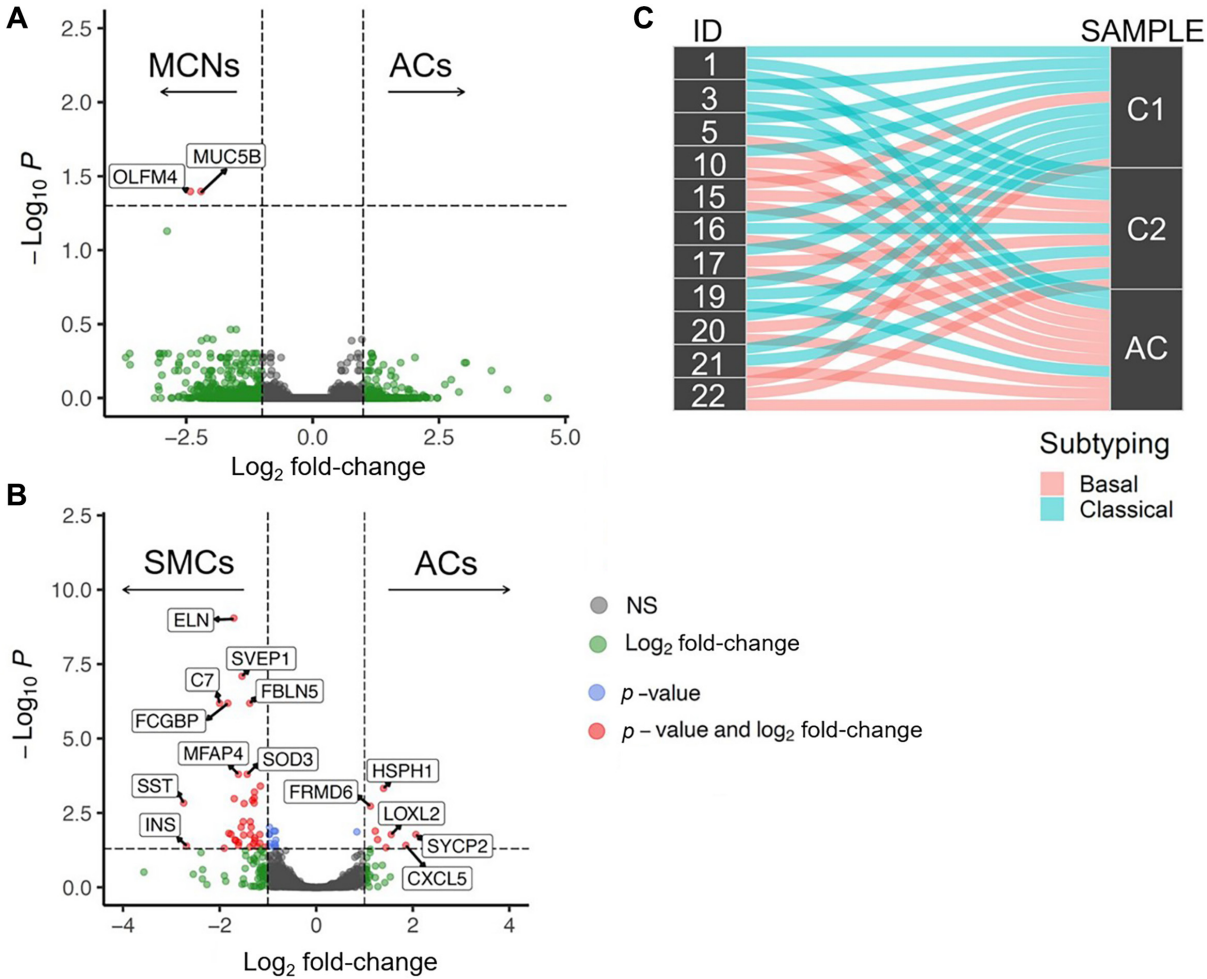
Summarizing figures related to transcriptome analyses. (A, B) Volcano plot showing the differentially expressed genes between (A) mucinous cystic neoplasms (MCNs) and associated carcinomas (ACs), and (B) between simple mucinous cysts (SMCs) and associated carcinomas (ACs). Red dots represent genes whose differential expression reached statistical significance (NS, not statistically significant). (C) Alluvial plot showing the subtype transcriptome classification and highlighting, in most cases, the transition from classical to basal phenotype during the progression to invasive adenocarcinoma.

Differential gene expression analysis comparing MCNs with their associated invasive carcinomas identified two significantly overexpressed genes in MCNs: *OLFM4* and *MUC5B* (Figure 3A). In contrast, differential gene expression analysis between SMCs and their associated invasive carcinomas revealed 49 genes significantly overexpressed in SMCs, including *ADAMTS1*, *ADAMTSL2*, *ADH1B*, *ALDH1A1*, *C3*, *C7*, *CCDC80*, *CCN2*, *CHRDL1*, *COL14A1*, *CRISPLD2*, *CSRNP1*, *DPT*, *EBF1*, *ELN*, *ERO1B*, *FBLN5*, *FCGBP*, *FOS*, *GPX3*, *HEYL*, *HSPB6*, *IGFBP2*, *INMT*, *INS*, *ITGBL1*, *KANK2*, *LDLRAD4*, *METTL7A*, *MFAP4*, *NFASC*, *NFIX*, *NUDT16*, *PDGFRA*, *PKD1P1*, *PRELP*, *PREX2*, *PTPRS*, *QSER1*, *SLC30A8*, *SMOC2*, *SOD3*, *SORBS2*, *SP4*, *SST*, *SVEP1*, *THSD4*, *ZBTB16*, and *ZFP36*, and eight genes significantly overexpressed in the invasive carcinomas, including *CXCL5*, *FRMD6*, *HSPA1B*, *HSPH1*, *LOXL2*, *MMP11*, *NEB*, and *SYCP2* (Figure 3B). Details regarding the differentially expressed genes between the different areas of SMCs (C1 versus C2 versus AC) are provided in the supplementary material, Table S3.

Transcriptome subtype analysis classified LGD cystic areas (C1) as classical in nine cases and basal in two cases, HGD cystic areas (C2) as classical in six cases and basal in five cases, and invasive cancers as classical in three cases and basal in eight cases. A classical to basal transition was observed in six cases as the disease progressed from cystic areas with LGD through cystic areas with HGD to invasive carcinomas. Three cases showed the basal subtype only in the cancer‐adjacent cystic area with HGD (C2) and invasive cancer, whereas the other three cases showed the basal subtype exclusively in the invasive component (Figure 3C).

### Comparative and survival analysis

A cohort of 85 MCNs without an associated invasive carcinoma was collected (supplementary material, Table S4), of which 78 had LGD, and 7 had HGD. Comparing all clinicopathological variables between MCNs with and without an associated invasive carcinoma (Student's *t*‐test), patients with MCNs with LGD were younger than those with HGD (*p* = 0.0002) and those with an associated invasive carcinoma (*p* < 0.0001). Furthermore, the mean cyst size of MCNs with LGD was smaller than that of MCNs with HGD (*p* = 0.0195), and that of MCNs with an associated invasive carcinoma (*p* = 0.0007). All the other comparisons did not reach statistical significance.

Univariate survival analysis across all cases identified male sex (hazard ratio [HR] = 17.24, 95% confidence interval [CI]: 1.70–174.99, *p* = 0.02) and tumor stage ≥2 (HR = 12.36, 95% CI: 1.25–121.79, *p* = 0.03) as significant risk factors for cancer‐specific mortality. Kaplan–Meier curves illustrate these findings (supplementary material, Figure S6). Tumor location in the pancreas showed a trend toward increased risk, although it did not reach statistical significance (HR = 5.43, 95% CI: 0.90–32.89, *p* = 0.07). No significant associations were observed in the multivariate model.

### NGS, FISH, and IHC

*CDKN2A*/*B* and *BRCA1* mutations were confirmed to be somatic, as no alterations were found in normal tissues. FISH for *CDKN2A* revealed a deletion of the second allele in cases 1 and 3, in which only one mutation was detected by DNA sequencing. IHC for Her2 confirmed the *ERBB2* amplification detected by NGS in the same regions (C1 and C2 of case no. 5, and C1, C2, and AC of case no. 13), showing diffuse and strong membrane positivity for Her2 in those regions. All SMCs showed no IHC positivity for estrogen receptor, progesterone receptor, and alpha‐inhibin. The results of IHC on mucins are summarized in supplementary material, Table S5. The most commonly expressed mucins were MUC5Ac in MCNs and MUC6 in SMCs. However, there were no statistically significant differences in terms of mean scores of mucins expression between MCNs and SMCs (Student's *t*‐test), also considering LGD versus HGD. Then, we specifically extracted the findings of transcriptome analysis, plotting the expression of mucins‐genes as a heatmap (supplementary material, Figure S7). The comparison of the transcriptome with IHC showed a linear correspondence between the two analyses, with positive cases at IHC showing positive values on gene expression profiles, and *vice versa*.

## Discussion

This study comprehensively characterized MCNs and SMCs of the pancreas and their concomitant invasive carcinomas. The key findings are as follows: (1) MCNs and SMCs are precursors of pancreatic cancer. For the first time, our data showed that SMC is a true precursor of pancreatic cancer, with molecular evidence linking it to invasive carcinoma. MCN was also confirmed as a precursor lesion of pancreatic cancer, with new evidence obtained from multiregional sequencing. (2) SMC‐derived carcinomas are aggressive in nature. Carcinomas originating from SMCs were more aggressive than those derived from MCNs, as evidenced by higher disease stages and greater prevalence of nodal metastases. (3) MCNs and SMCs display genetic similarities to PDAC. Both MCNs and SMCs share a mutational profile similar to that of conventional PDAC, with *KRAS*, *TP53*, and *CDKN2A* being the most commonly altered genes. (4) Intracystic heterogeneity. While most molecular alterations were present in both analyzed cystic areas, *RNF43* showed the highest heterogeneity. (5) Genetic drivers of tumor progression. The invasive component had the highest prevalence of molecular alterations, with *CDKN2A* alterations typically restricted to invasive cancers in the MCN cohort. It may be used as a marker of malignant transformation in this tumor category. (6) Classic‐to‐basal subtype switch in RNA‐seq. Most cases show a transcriptomic switch from the classical to the basal subtype during the progression from cystic lesions to invasive carcinomas. This transition aligns with findings in other pancreatic precursor lesions and suggests a common mechanism in pancreatic tumorigenesis. (7) Survival moderators. Survival analysis showed male sex and tumor stage ≥2 as risk factors for cancer‐specific mortality.

A critical finding of the molecular investigation came from the comparative analysis of mucinous cysts and their concomitant adenocarcinomas. For the first time these data definitively established SMC as the origin of invasive pancreatic cancer and provided new evidence in confirming this role for MCN. Prior to this investigation, the role of SMC in pancreatic tumorigenesis had been hypothesized, but has never been conclusively demonstrated. It has been suggested that they are a neoplastic entity based on the possible presence of HGD and *KRAS* mutations in some cases, but to date no SMCs progressing to invasive cancer have been documented [14, 15, 16]. The current study demonstrates for the first time the histological and molecular association between SMC and concomitant carcinoma: this entity can now be considered and classified as a true precursor of pancreatic cancer.

It is worth noting that the definition and diagnosis of SMC require some critical considerations. First, a late‐stage (or end‐stage) of an MCN, where the ovarian‐type stroma has extensively regressed, should be excluded. In our series, all the SMCs were entirely sampled and submitted for histological analysis, and no ovarian‐type stromal cells were detected. Of note was the IHC for estrogen receptor, progesterone receptor, and alpha‐inhibin, which showed no ovarian‐type stromal cells. Furthermore, other types of cysts potentially lined by a mucinous epithelium should be excluded. Along this line, the first step is to exclude IPMNs: this distinction is based on the lack of papillary formations and communication with the pancreatic ductal tree. Of note, none of our SMCs showed papillae, and they did not communicate with ducts. Moreover, some cysts with peculiar features that can be lined by mucinous epithelium were also excluded based on histological analysis, such as the ciliated foregut cysts [33]. In the context of a differential diagnosis, a potentially controversial point can also be found in distinguishing SMCs from retention cysts, above all for those cystic lesions concomitant with invasive adenocarcinomas. Indeed, it is important to exclude the presence of an adenocarcinoma arising inside a retention cyst, giving rise to aspects of cancerization. In our series, however, this eventuality appears very unlikely, since we found the same molecular alterations also in areas with LGD of the cysts, which cannot represent an area of cancerization, where usually an abrupt transition from LGD to HGD/atypia is observed.

Remarkably, cancers derived from SMCs may be potentially more aggressive than those derived from MCNs. Indeed, they show higher tumor stages and a higher rate of nodal metastasis at the time of diagnosis. Although these findings are based on a few cases and should be validated by future studies on this topic, they may further highlight the presence of clinicopathological differences between MCNs and SMCs. Regarding other clinicopathological data, we observed statistically significant differences in terms of patients' age and cysts' size between MCNs with LGD versus patients with MCNs with HGD and MCNs with an associated invasive carcinoma. This finding is a confirmatory result of previous investigations on the natural history of MCNs, which reported similar differences along this line [34, 35]. Concerning the clear correlation between co‐occurring mucinous cysts and associated carcinoma in the vast majority of cases (95.7%), this finding appears to be different from that of IPMN, where a non‐negligible fraction of concomitant adenocarcinomas (almost 20%) were independent of intraductal lesions [36, 37, 38]. This finding further highlights the differences between MCNs and SMCs versus IPMN. In this complex scenario, it should be acknowledged that MCNs and SMCs may have different origins. Indeed, recent studies have clarified that MCNs likely derive from dysembryogenic residues attributable to ovarian tissue [12, 39, 40], as also supported by the presence of ovarian stroma as a distinctive histological feature of these lesions. Conversely, SMCs lack this feature, and their origin is still unclear. Based on the morphology and previous observations on the epithelial lining [15, 40], one hypothesis is that they may represent a larger version of PanINs, but further studies are needed along this line to elucidate this fascinating topic.

Regarding the results of DNA‐NGS of mucinous cysts and associated invasive cancers, it is crucial to highlight the significant similarities between these lesions and conventional PDAC, with the addition of *RNF43*. Overall, the prevalence of genetic alterations recapitulated the molecular landscape of conventional PDAC. The most commonly altered genes were indeed *KRAS* (91%) and *TP53* (55%). Our study highlighted the clinical implications of *CDKN2A* alterations. They are predominantly observed in invasive carcinomas, suggesting that *CDKN2A* may serve as a marker of malignant transformation in mucinous cysts. This finding has potential clinical implications, as it could aid in the stratification of patients at a higher risk of developing invasive cancer. Furthermore, the presence of *CDKN2A* alterations may also be assessed in the fluid of mucinous cysts of the pancreas, opening new interesting opportunities for novel algorithms of precision medicine in these patients, also in the preoperative setting. Along this line, a recent molecular investigation on pancreatic cyst fluid already described *CDKN2A* alterations as a marker of advanced pancreatic neoplasia [41]. Notably, gene mutations usually considered late events in pancreatic oncogenesis, such as those affecting *TP53* and *SMAD4*, have also been found in noninvasive cystic areas. Along this line, *SMAD4* mutations, quite common in PDAC (around 50% of cases), were rarely observed in our cohort of mucinous cysts and associated adenocarcinomas (14% of cases). A similar low prevalence (8%) of *SMAD4* variations was already reported for SMCs in a recent study [42]. Regarding MCNs, the prevalence of *SMAD4* alterations is reported as very low in the noninvasive component, but higher in the associated invasive carcinoma [43]. Based on the current study, which represents the largest series of MCNs analyzed with multiregional NGS, we can also add to this knowledge that *CDKN2A* may play a significant role in the carcinogenesis of MCNs. Interestingly, the observed intracystic heterogeneity was limited when compared to that of IPMN, where recent studies showed that even early‐stage lesions contained multiple independent clones, each with distinct mutations, thus showing a multiclonal origin [44]. These findings suggest a more linear evolutionary progression in mucinous cysts compared to the more complex multiclonal origin seen in IPMNs.

Although this is a rare finding, it may be of interest to highlight the presence of potentially actionable alterations in invasive cancers in the analyzed cohort, including *BRAF* mutations and *ERBB2* amplification (in two different cases). Notably, *ERBB2* was amplified in one additional case, but the amplification was restricted to the noninvasive component, thus without any significance in terms of tailored treatments. This finding underscores the complexity of tumor evolution and the central role of pathologists in selecting the appropriate material for molecular analysis to correctly inform therapeutic decisions.

Other interesting considerations can be made based on RNAseq findings. Specifically, it is critical to highlight the recurrent presence of a switch from the classical to the more aggressive basal transcriptome subtype during progression in most cases. This finding can be seen in the light of recent studies, which found evidence for the first time of transcriptome plasticity during pancreatic cancer progression and metastasis, and across different stages of the disease [45, 46, 47]. Indeed, transcriptome profiles of PDAC and related precursors should be seen as a changing landscape. Notably, transcriptome plasticity has also been described for other well‐established precursors of pancreatic cancer, such as IPMNs and ITPNs, where the classical subtype has been more commonly detected in preinvasive lesions and the basal subtype is more commonly found in the invasive component [35, 48, 49, 50]. Such data have also been recently confirmed by spatial transcriptomics [50]. Thus, this classical‐to‐basal switch appears to be a biological mechanism shared by different macroscopic precursor lesions of the pancreas.

This study has some limitations. First, molecular analysis did not encompass the entire genome, which may have led to the omission of potentially significant molecular events. However, the CORE panel that we adopted was derived from previously reported whole‐genome sequencing studies that focused on clinically relevant alterations. Furthermore, in some samples, particularly those from cystic areas, neoplastic cellularity was low owing to the intrinsic features of the tumors, which were sometimes composed of a monolayered epithelium. In these samples, assessment of copy number variation was not always possible. However, specific FISH assays were employed to address this issue for key alterations, such as those involving *CDKN2A*. Finally, although the number of SMCs was relatively small, it was sufficient to suggest SMCs as precursors of pancreatic cancer. Along this line, further studies investigating the histomolecular profiles of SMCs and associated adenocarcinomas are needed to better characterize the natural history of these lesions as a precursor of pancreatic cancer, ideally presenting larger cohorts of cases (also without an associated adenocarcinoma and differentiating lesions with LGD and HGD) with complete clinicopathological data.

In conclusion, we present an integrative clinicopathological and molecular characterization of a series of pancreatic mucinous cysts and associated adenocarcinomas. We demonstrated that SMCs are *bona‐fide* precursors of pancreatic cancer and provide novel insights into the malignant transformation of MCNs. *CDKN2A* alterations, predominantly occurring in the invasive component, may act as drivers of tumor invasion and serve as potential biomarkers for identifying high‐risk cysts. Globally, these data provide critical insights into pancreatic tumorigenesis with potential immediate implications for tumor taxonomy and clinical management.

## Author contributions statement

Study conception and design was provided by CL. Original material for the study was provided by AP, MSA, JSS, ALJ, GM, JV, LAAB, LDW, AJG, DKC, RS, AS and CL. Clinical analysis was done by AP, DF, PM, JV, GM, MM, LAAB, LDW, AJG, DKC, RDB, MDO, RS, AS and CL. Histological analysis was performed by LAAB, JV, LDW, AJG, AS and CL. Molecular analysis was performed by AP, MB, AG, DP, AM, SG, MS, CS, PM, NS, ALJ, DKC, VC, RTL, AS and CL. The manuscript was written by AP and CL. All authors contributed to the final editing and gave approval of the final version.

## Supporting information


Supplementary materials and methods
**Figure S1.** Highly‐illustrative histological features of mucinous cystic neoplasms of the pancreas
**Figure S2.** Highly‐illustrative histological features at low magnification of the four cases of simple mucinous cysts of the pancreas of this study
**Figure S3.** Highly‐illustrative histological features at high magnification of the four cases of simple mucinous cysts of the pancreas of this study
**Figure S4.** Representative images of three cases of simple mucinous cysts of this case series with CT imaging (for one case, case #19, imaging was not available)
**Figure S5.** Summarizing diagram showing intratumor heterogeneity based on DNA next‐generation sequencing (on the x‐axis: total number of cases)
**Figure S6.** Kaplan–Meier curves based on (A) sex and (B) tumor stage of the patients in the current study
**Figure S7.** Heatmap showing the normalized expression Z‐scores of the selected genes of mucins across samples, with annotations for case, type, and condition (C1, low‐grade dysplasia; C2, high‐grade dysplasia)
**Table S1.** Targeted genes in the CORE sequencing assay
**Table S2.** Summary of the chromosomal alterations detected in the current case‐series
**Table S3.** Differential gene expression analysis (number of overexpressed genes) presented by the different tumor components of SMC
**Table S4.** Summarizing table of a cohort of mucinous cystic neoplasms without an associated invasive carcinoma
**Table S5.** Immunohistochemical scores of mucins in the current case series

## Data Availability

Data are available in the article and in the supplementary material. Data are also available from the corresponding author upon reasonable request.
