# Supplementary material for: Mucinous cystic neoplasms and simple mucinous cysts are two distinct precursors of pancreatic cancer: clinicopathological, genomic, and transcriptomic characterization
Source: J Pathol. 2025 May 15;266(4-5):421–34. doi: 10.1002/path.6437 (PMC12256392; doi:10.1002/path.6437)
Supplement: Supplementary file 1 — Supplementary materials and methods Figure S1. Highly‐illustrative histological features of mucinous cystic neoplasms of the pancreas Figure S2. Highly‐illustrative histological features at low magnification of the four cases of simple mucinous cysts of the pancreas of this study Figure S3. Highly‐illustrative histological features at high magnification of the four cases of simple mucinous cysts of the pancreas of this study Figure S4. Representative images of three cases of simple mucinous cysts of this case series with CT imaging (for one case, case #19, imaging was not available) Figure S5. Summarizing diagram showing intratumor heterogeneity based on DNA next‐generation sequencing (on the x‐axis: total number of cases) Figure S6. Kaplan–Meier curves based on (A) sex and (B) tumor stage of the patients in the current study Figure S7. Heatmap showing the normalized expression Z‐scores of the selected genes of mucins across samples, with annotations for case, type, and condition (C1, low‐grade dysplasia; C2, high‐grade dysplasia) Table S1. Targeted genes in the CORE sequencing assay Table S2. Summary of the chromosomal alterations detected in the current case‐series Table S3. Differential gene expression analysis (number of overexpressed genes) presented by the different tumor components of SMC Table S4. Summarizing table of a cohort of mucinous cystic neoplasms without an associated invasive carcinoma Table S5. Immunohistochemical scores of mucins in the current case series [file PATH-266-421-s001.docx]

**Mucinous cystic neoplasms and simple mucinous cysts are two distinct precursors of pancreatic cancer: clinicopathological, genomic, and transcriptomic characterization**

A Pea, M Bevere, A Gkountakos, D Pasini *et al.* *J Pathol* <https://doi.org/10.1002/path.6437>

**Supplementary materials and methods**

**Supplementary Figures S1–S7**

**Supplementary Tables S1–S5**

Reference numbers refer to the main text list.

**Supplementary materials and methods**

**Multiregional DNA sequencing**

Sequencing was performed using NextSeq 500 (Illumina, San Diego, CA, USA). In brief, CORE panel analysis started with demultiplexing performed with FASTQ Generation v1.0.0 on the BaseSpace Sequence Hub (https://basespace.illumina.com, last accessed 09/30/2024). Mapped reads were subjected to PCR duplication removal and indexed using biobambam2 v2.0.146 (https://gitlab.com/german.tischler/biobambam2.git; last accessed 09/30/2024) [51]. Single nucleotide variants were identified using shearwater [52]. Small (< 200 bp) insertions and deletions were identified using Pindel version 0.2.5b8 [53]. Copy number alterations of targeted genes were detected using GeneCN software (https://github.com/wwcrc/geneCN; last accessed 09/30/2024). Structural rearrangements were detected using BRASS software [54]. Tumor variants were classified as benign (class 1), likely benign (class 2), variant of uncertain significance (VUS class 3), likely pathogenic (class 4), or pathogenic (class 5), following the guidelines of the American College of Medical Genetics and Genomics and the Association for Molecular Pathology (ACMG/AMP) [55].

**Multiregional RNA sequencing**

For RNA sequencing, RSEM transcript quantification was imported in R through the tximport package v4.0, and raw counts were normalized using the R/Bioconductor package DESeq2 [56,57]. Data processing and differential gene expression analysis were performed using DESeq2 [57]. GSVA R package was used to calculate the main pancreatic ductal adenocarcinoma transcriptomics subtype gene set scores using the ssGSEA method [58,59].

**Fluorescent *in situ* hybridization**

In cases where *CDKN2A* was altered by mutation in one allele but copy number variation analysis was not possible due to a relatively low cancer cellularity, specific fluorescence *in situ* hybridization (FISH) for *CDKN2A* was performed on FFPE sections using a commercially available probe (ZytoLight, CDKN2A/CEN 9, ZytoVision, Bremerhaven, Germany). The probe was designed to detect *CDKN2A* deletions (chromosomal region 9p21.3). The analysis was conducted following the manufacturer's protocols.

**Immunohistochemistry (IHC) for mucins**

Additional IHC was performed on all cysts to investigate the expression of the most important pancreatic mucins, namely MUC1, MUC2, MUC4. MUC5AC, and MUC6, using the following antibodies: MUC1 (Ma695; prediluted; Leica, Wetzlar, Germany), MUC2 (Ccp58; prediluted; Novocastra, Buffalo Grove, IL, USA), MUC4 (CB12; 1:3,000; Abcam, Cambridge, UK,), MUC5A5 (CLH2; 1:50; Dako, Jena, Germany), and MUC6 (CLH5; prediluted; Leica). The overall evaluation used a combined quantitative and qualitative score. First, the percentage of positive cells was calculated by assigning a quantitative score as follows: no positive cells = score 0, 1–25% = 1, 26–50% = 2, 51–75% = 3, 76–100% = 4. Then, a qualitative evaluation was performed, assigning a score based on the staining intensity: score 0 = no staining, 1 = weak staining, 2 = moderate staining, and 3 = strong staining. Finally, the combined score was calculated by multiplying the quantitative and qualitative scores, with the final combined score ranging from 0 to 12.

**Survival analysis**

Survival analysis started with univariate Cox regression analyses, which were performed to investigate the association between the clinicopathological and molecular data and survival indices. The outcomes considered were the cancer-specific and disease-free survival rates. Data from Cox regression analyses were graphically reported using Kaplan–Meier curves. The results are presented as hazard ratios (HRs) with 95% confidence intervals (CIs). A multivariate model was built using factors potentially associated with the outcomes of interest, taking a *p*-value < 0.10 as the inclusion criterion. All analyses were performed using SPSS (Chicago, IL, USA), 26.0 software.

**
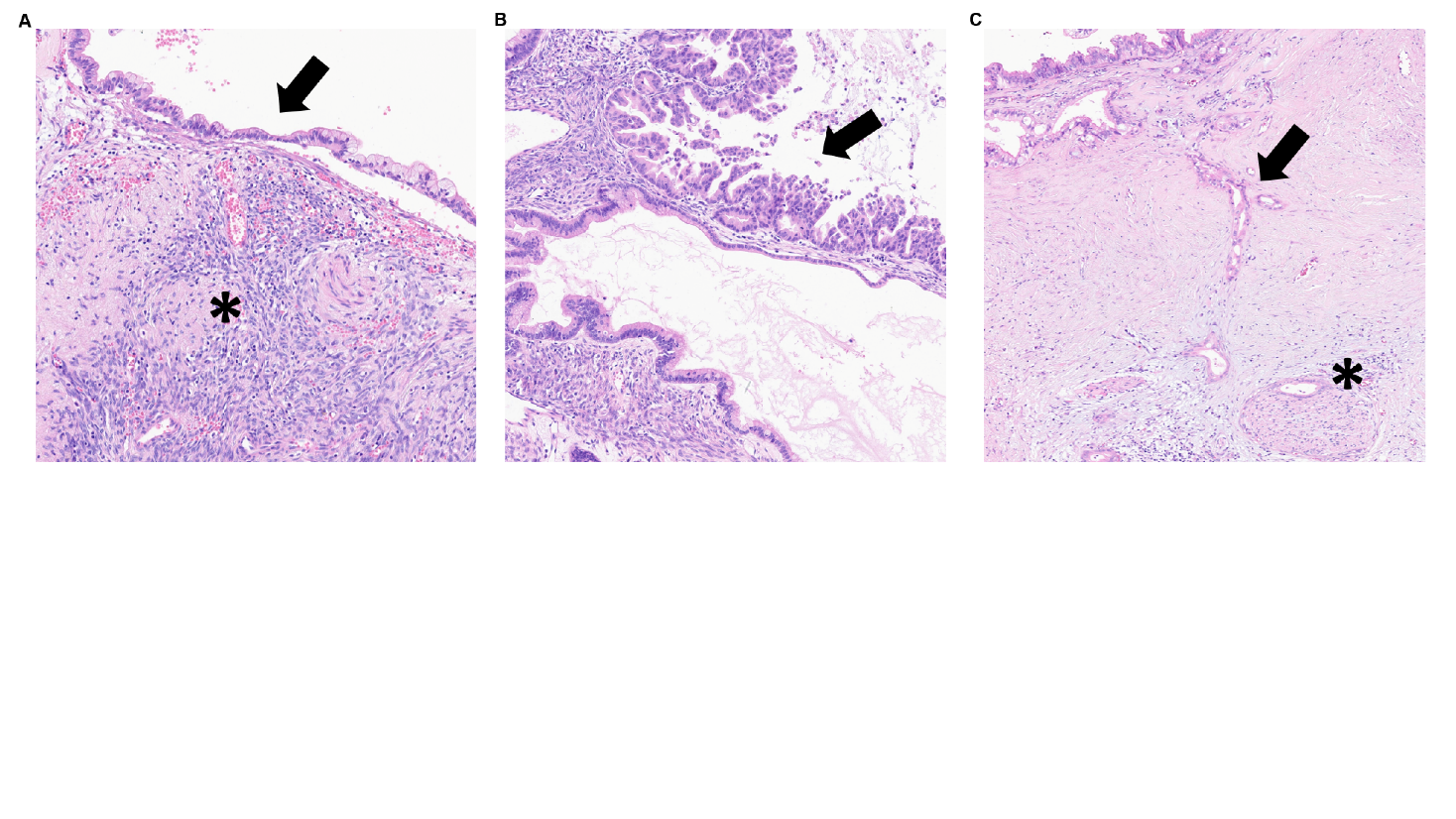
**

**Figure S1.** **Highly-illustrative histological features of mucinous cystic neoplasms of the pancreas.** (A) Area with low-grade dysplasia: the mucinous epithelium with low-grade dysplasia is visible (arrow) with the subepithelial ovarian-type and hypercellular stroma (asterisk), which is typical of this entity (hematoxylin-eosin staining, original magnification 20×). (B) Region with high-grade dysplasia (arrow; 20×). (C) Area with the associated invasive adenocarcinoma (arrow), which infiltrates also a nerve (peri-neural invasion, asterisk; 10×). Highly-illustrative histological features of mucinous cystic neoplasms of the pancreas.

**
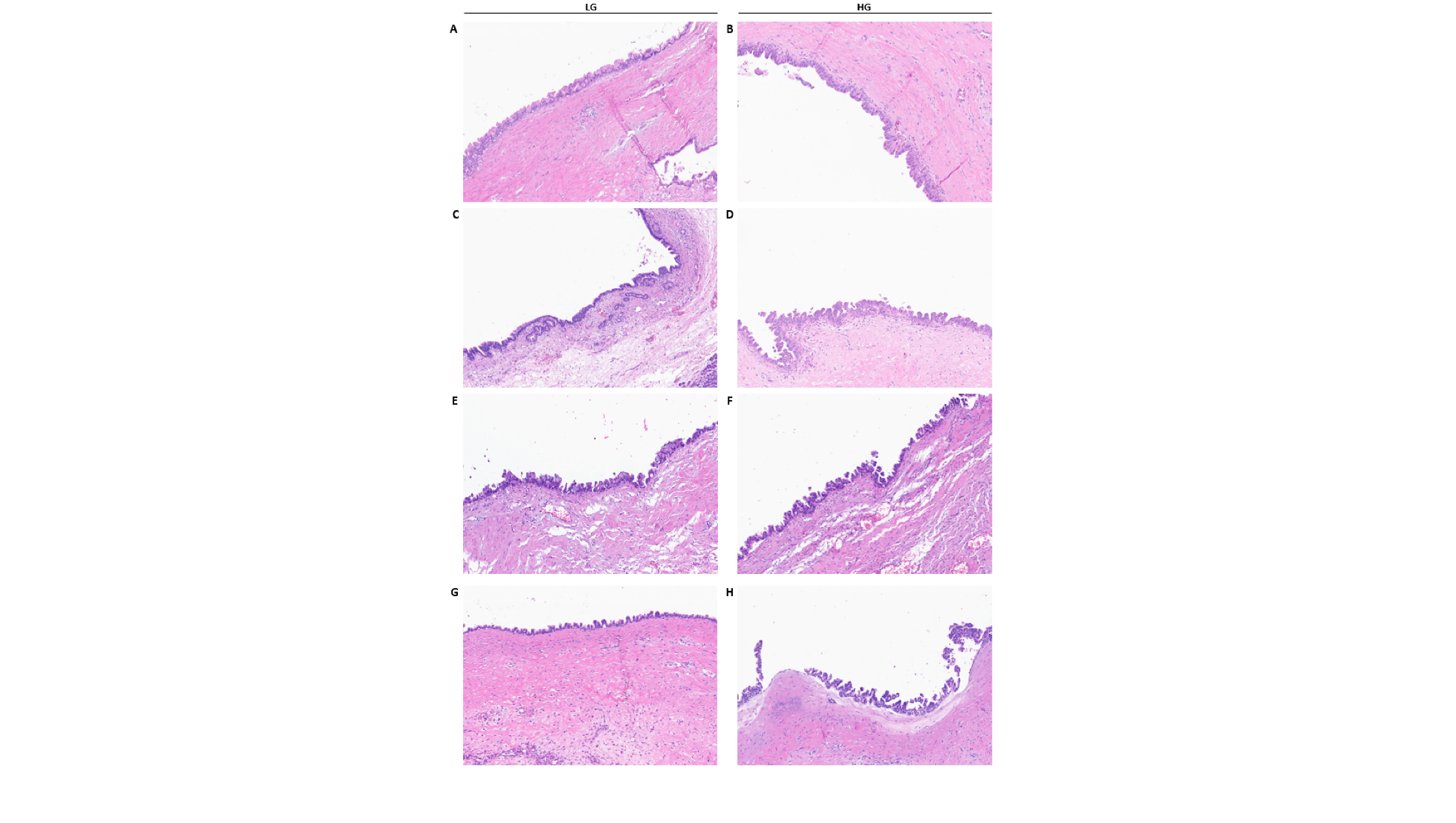
**

**Figure S2.** **Highly-illustrative histological features at low magnification of the four cases of simple mucinous cysts of the pancreas of this study.** (A) Case #19, area with low-grade dysplasia (hematoxylin-eosin staining, original magnification 4×). (B) Case #19, area with high-grade dysplasia (4×). (C) Case #20, area with low-grade dysplasia (4×). (D) Case #20, area with high-grade dysplasia (4×); (E) Case #21, area with low-grade dysplasia (4×). (F) Case #21, area with high-grade dysplasia (4×). (G) Case #22, area with low-grade dysplasia (4×). (H) Case #22, area with high-grade dysplasia (4×).

**
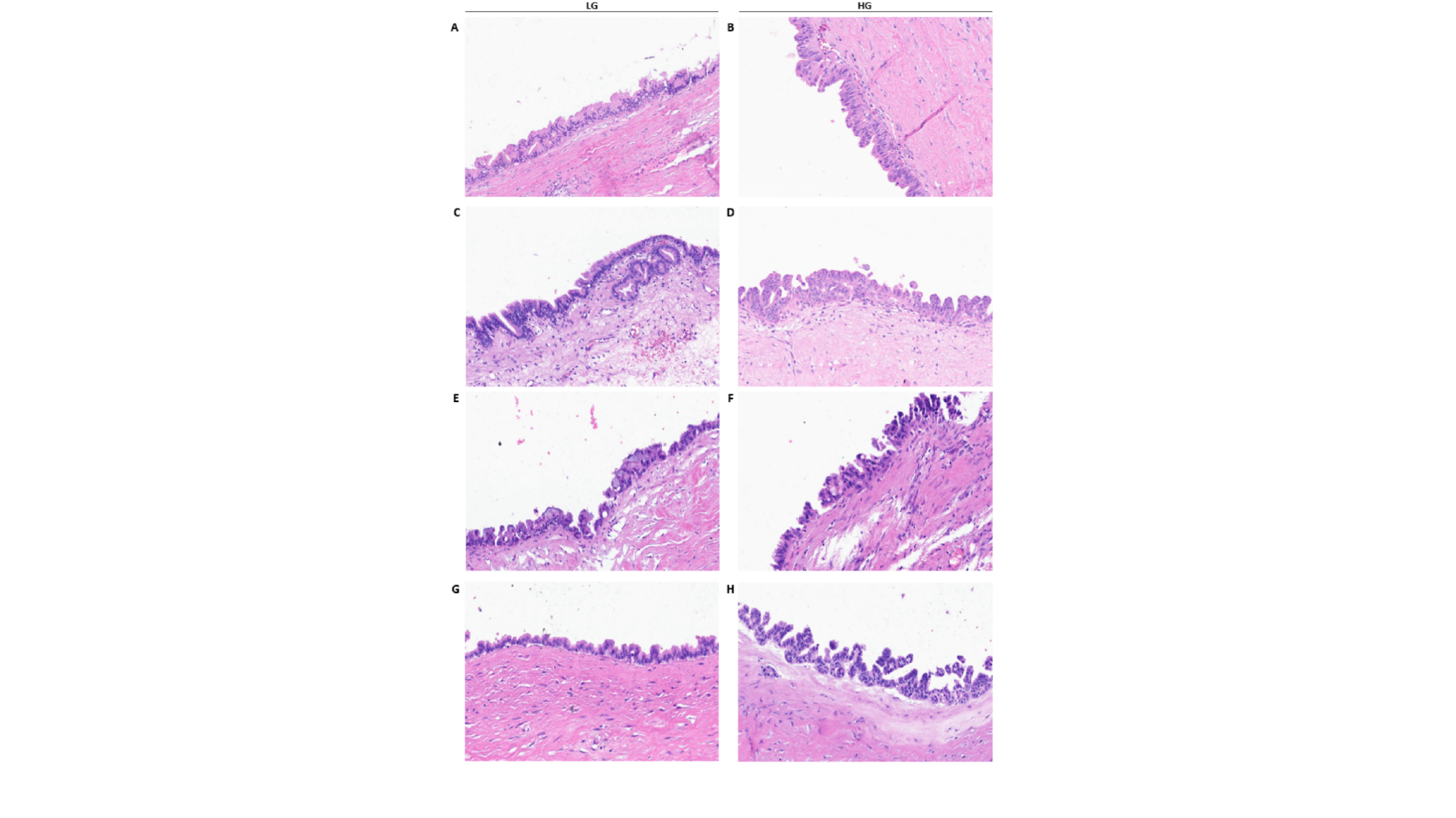
****Figure S3.** **Highly-illustrative histological features at high magnification of the four cases of simple mucinous cysts of the pancreas of this study (LG: low-grade dysplasia; HG: high-grade dysplasia).** (A) Case #19, area with low-grade dysplasia (Hematoxylin-eosin staining, original magnification 20×). (B) Case #19, area with high-grade dysplasia (20×). (C) Case #20, area with low-grade dysplasia (20×). (D) Case #20, area with high-grade dysplasia (20×). (E) Case #21, area with low-grade dysplasia (20×). (F) Case #21, area with high-grade dysplasia (20×). (G) Case #22, area with low-grade dysplasia (20×). (H) Case #22, area with high-grade dysplasia (20×).

**
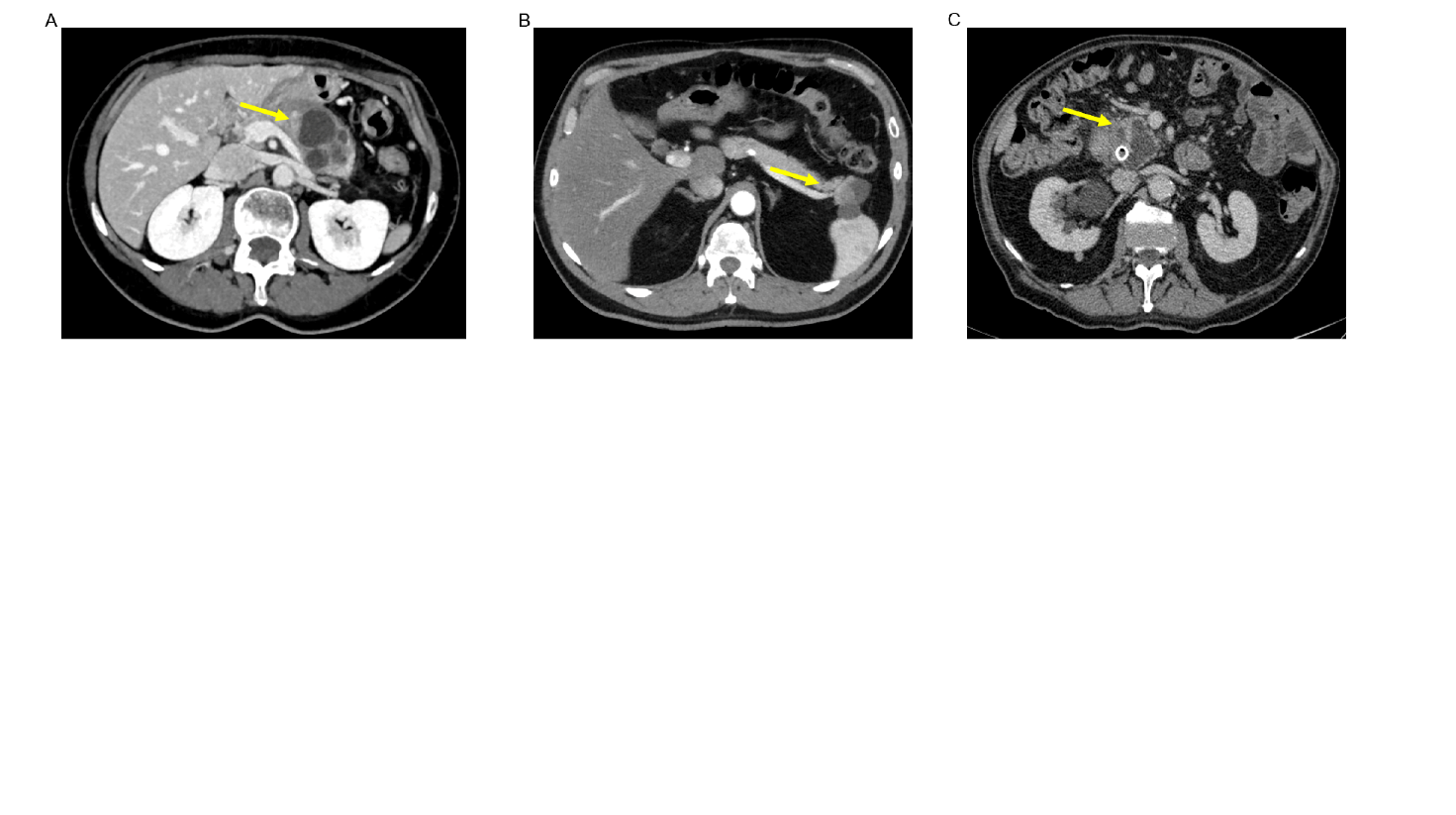
**

**Figure S4.** **Representative images of three cases of simple mucinous cysts of this case series with computed tomography (CT) imaging (for one case, case #19, imaging was not available).** The three images show multilobulated cystic lesions in (A) case #21; (B) case #22; (C) case #20 with internal septations and a solid component. While these features are not typical of retention cysts, careful evaluation also based on histology is required for differential diagnosis of pancreatic cystic lesions.

**
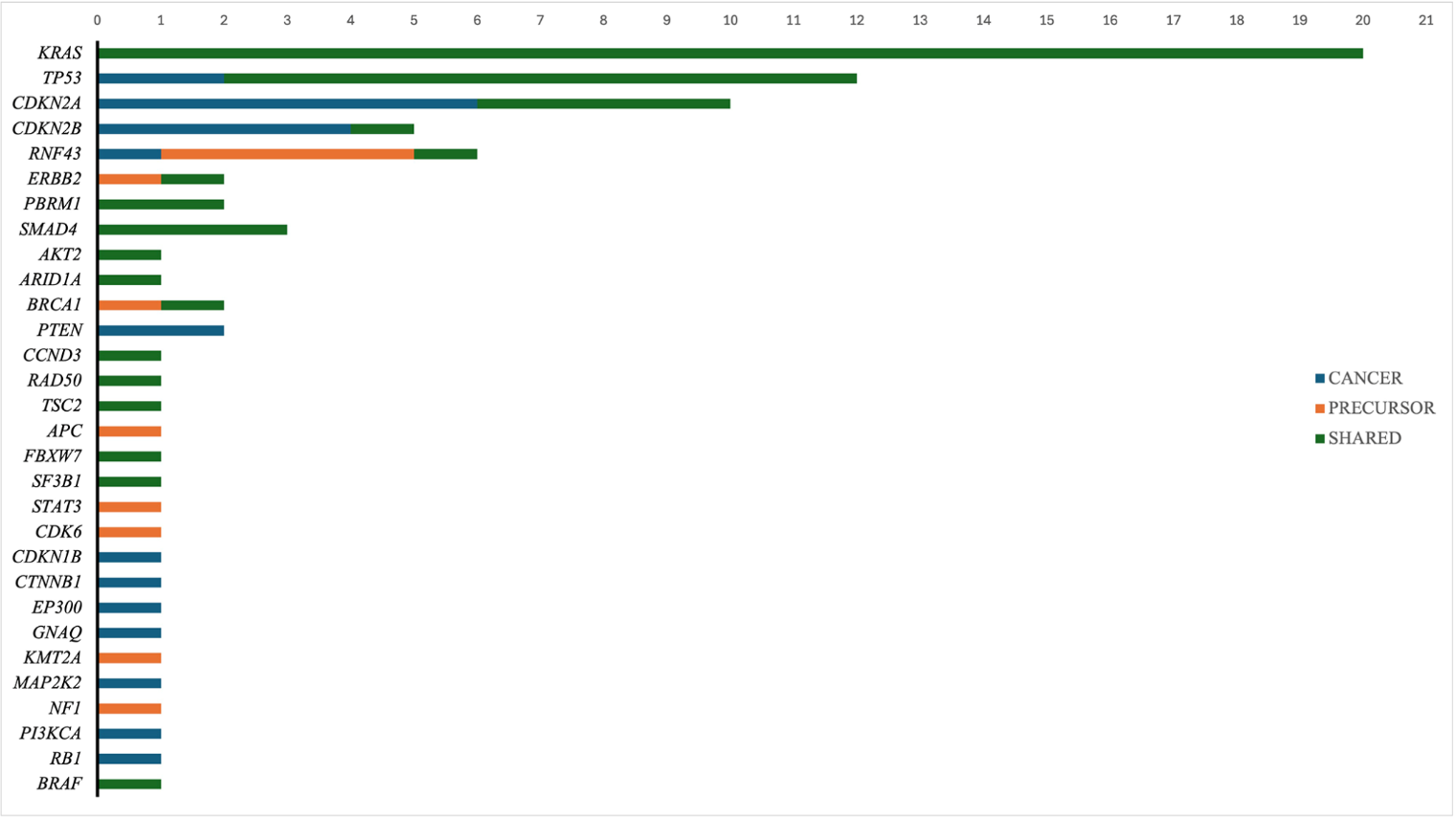
**

**Figure S5. Summarizing diagram showing intratumor heterogeneity based on DNA next-generation sequencing (on the x-axis: total number of cases).** Green color: molecular alterations shared by the mucinous cyst (at least one area) and the matched associated carcinoma; blue: alterations restricted to the invasive component; orange: alterations restricted to the mucinous cyst (at least one area). More specifically, the distribution of the genetic alterations and the relevant clinical significance is the following: *KRAS*: 20/20 missense mutation (pathogenic); *TP53*: 7/12 missense mutation (pathogenic), 2/12 splice site mutation (pathogenic), 1/12 splice site mutation (likely pathogenic), 2/12 frameshift insertion (pathogenic); *CDKN2A*: 4/10 homozygous deletion (pathogenic), 1/10 frameshift deletion (pathogenic), 1/10 missense mutation (pathogenic), 1/10 missense mutation (likely pathogenic), 2/10 stop gain mutation (pathogenic), 1/10 frameshift insertion (likely pathogenic); *CDKN2B*: 4/5 homozygous deletion (pathogenic), 1/5 LOH (likely pathogenic); *RNF43*: 4/6 stop gain mutation (either pathogenic or likely pathogenic), 1/6 splice site mutation, 1/6 LOH (likely pathogenic); *ERBB2*: 2/2 gene amplification (pathogenic); *PBRM1*: 1/2 missense mutation (pathogenic), 1/2 stop gain mutation (likely pathogenic); *SMAD4*: 2/3 frameshift deletion (likely pathogenic), 1/3 LOH (likely pathogenic); *AKT2*: 1/1 gene amplification (pathogenic); *ARID1A*: 1/1 frameshift insertion (likely pathogenic); *BRCA1*: 2/2 frameshift deletion (pathogenic); *PTEN*: 2/2 LOH (likely pathogenic); *CCND3*: 1/1 gene amplification (pathogenic); *RAD50*: 1/1 complex mutation (likely pathogenic); *TSC2*: 1/1 frameshift deletion (likely pathogenic); *APC*: 1/1 frameshift insertion (pathogenic), *FBXW7*: 1/1 missense mutation (pathogenic); *SF3B1*: 1/1 missense mutation (pathogenic), *STAT3*: 1/1 gene amplification (VUS); *CDK6*: 1/1 gene amplification (pathogenic), *CDKN1B*: 1/1 homozygous deletion (pathogenic); *CTNNB1*: 1/1 missense mutation (pathogenic), *EP300*: 1/1 missense mutation (likely pathogenic); *GNAQ*: 1/1 missense mutation (pathogenic); *KMT2A*: 1/1 frameshift deletion (likely pathogenic); *MAP2K2*: 1/1 missense mutation (pathogenic); *NF1*: 1/1 frameshift deletion (pathogenic); *PI3KCA*: 1/1 missense mutation (pathogenic); *RB1*: 1/1 missense mutation (likely pathogenic); *BRAF*: 1/1 missense mutation (pathogenic). Abbreviations: LOH, loss of heterozygosity; VUS, variant of unknown significance.

**
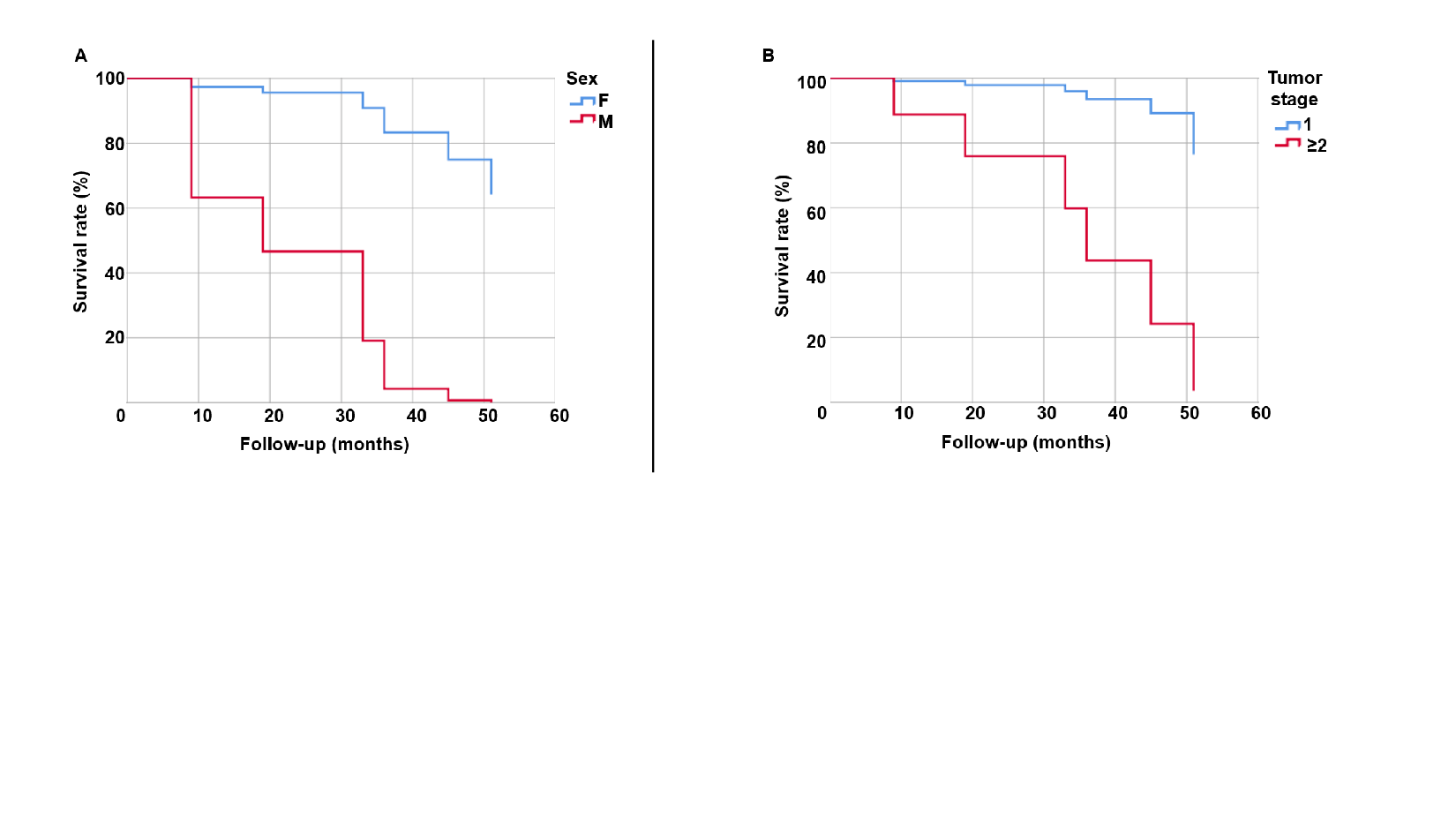
**

**Figure S6.** **Kaplan–Meier curves based on (A) sex and (B) tumor stage of the patients in the current study.** Male sex and tumor stage ≥ 2 are risk factors for cancer specific mortality.

**
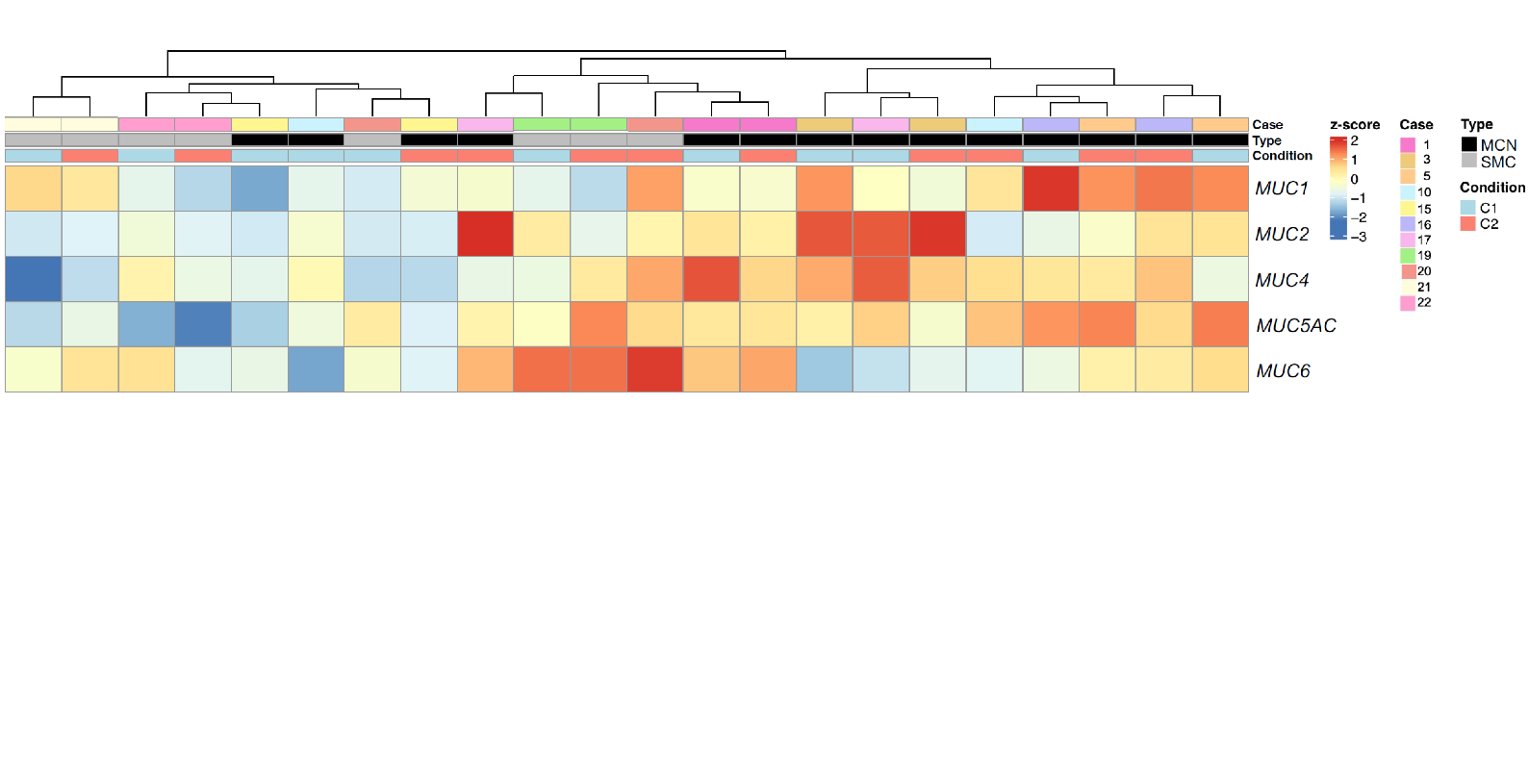
**

**Figure S7.** **Heatmap showing the normalized expression Z-scores of the selected genes of mucins across samples, with annotations for case, type, and condition (C1, low-grade dysplasia; C2, high-grade dysplasia).** Columns (samples) are grouped according to hierarchical clustering based on expression data.

**Table S1.** Targeted genes in the CORE sequencing assay

**Genes covered for all coding exons**

AKT1 AKT2 AKT3 ALK AMER1 APC APLNR AR ARAF ARID1A ARID1B ARID2 ASXL1 ATM ATR ATRX B2M BAP1 BLM BRAF BRCA1 BRCA2 CBL CCND1 CCND3 CD274 CD58 CDK12 CDK4 CDKN1A CDKN1B CDKN2A CDKN2B CHEK2 CIITA CREBBP CTCF CTNNB1 DAXX DICER1 DNMT3A EGFR EP300 EPHA3 ERBB2 ERBB3 ERBB4 ERG ESR1 ETV6 EZH2 FAS FBXW7 FGFR1 FGFR2 FGFR3 FGFR4 GATA3 GNA11 GNAQ GNAS H3F3A H3F3B HIST1H3B HIST1H3C HIST2H3C HLA-A HLA-B HLA-C HNF1A HRAS IDH1 IDH2 JAK1 JAK2 JAK3 JUN KDR KIT KLF4 KMT2A KRAS MAP2K1 MAP2K2 MAP2K4 MAP3K1 MAPK1 MAX MED12 MEN1 MET MLH1 MSH2 MSH6 MTOR MUTYH MYB MYC MYCN NBN NF1 NF2 NFE2L2 NOTCH1 NOTCH2 NOTCH3 NOTCH4 NPM1 NRAS NTRK1 PALB2 PBRM1 PDCD1LG2 PDGFRA PDGFRB PHF6 PIK3CA PIK3CB PIK3R1 PMS2 POLE POLQ PPP2R1A PTCH1 PTEN PTPN11 RAC1 RAD21 RAD50 RAF1 RB1 RET RHOA RNF43 ROS1 RPL5 RUNX1 SETBP1 SETD2 SF3B1 SMAD4 SMARCA4 SMARCB1 SMO SOCS1 SPOP STAG1 STAG2 STAT3 STAT5B STK11 SYK TGFBR2 TP53 TSC1 TSC2 U2AF1 VHL WT1

**Cancer CORE: genes covered for copy number alterations**

AKT1 AKT2 AKT3 ALK APC APLNR AR AURKA AXL B2M BCL2 BRAF BRCA1 BRCA2 CCND1 CCND2 CCND3 CCNE1 CD274 CDK2 CDK4 CDK6 CDKN1B CDKN2A CDKN2B CIITA CTNNB1 EGFR EPHA3 ERBB2 ERG ESR1 ETV6 EZH2 FGF19 FGFR1 FGFR2 FGFR3 FGFR4 HGF HLA-A HLA-B HLA-C IGF1R JAK1 JAK2 JAK3 JUN KIT KRAS MCL1 MDM2 MET MLH1 MYC NFE2L2 NOTCH1 NOTCH2 NOTCH3 NOTCH4 NTRK1 PALB2 PDCD1LG2 PDGFRA PDGFRB PIK3CB PTEN RAF1 RB1 RET SMAD4 SOCS1 SRC STAT3 SYK TERT TP53 TSC1 TSC2 VHL YAP1

**Cancer CORE: genes with full-gene footprint for structural variations**

APLNR B2M BRCA1 BRCA2 CDKN2A CDKN2B HLA-A HLA-B HLA-C PTEN RB1 TP53 TSC1

**Cancer CORE: genes covered for fusions detection**

ALK BRAF EGFR ETV6* FGFR2 FGFR3 NTRK1 RAF1 RET ROS1 TERT

Note: *ETV6 reports NTRK3 fusions.

**Table S2.** Summary of the chromosomal alterations detected in the current case-series

| **ID case** | **Sample** | **LOH** | **Copy–neutral LOH** | **Gain** |
| --- | --- | --- | --- | --- |
| 1 | C1 | chr5q14.3–q31.1, chr6p25.3–p22.1, chr17p13.3–p13.1, chr22q11.1–q12.1, chr22q12.2–q13.1, chrX | chr9 | chr2q31.3–q37.3 |
|  | C2 | N/A | N/A | N/A |
|  | AC | chr9, chr17p13.3–p13.1 |  | - |
| 5 | C1 | N/A | N/A | N/A |
|  | C2 | chr22p11.21–12.3 | chr17p13.3–q11.2 | chr22q13.1–13.2 |
|  | AC | chr9p24.3–13.3, chr12p13.33–13.2, chr16p13.3–12.2, chr17p13.3–q11.2, chr18p11.21–q23, chr22q11.1–12.2 | - | - |
| 7 | C1 | chr9p21.13–q21.32, chr11q13.4–q23.1 | - | - |
|  | C2 | chr9p21.13–q21.32, chr17q22–q25.1 | *-* | *-* |
|  | AC | chr9p21.13–q21.32 | - | chr20p12.3–q11.21 |
| 8 | C1 | chr9p21.3–p22.2 | - |  |
|  | C2 | chr7q22.1–q36.3, chr9p21.3–p22.2 | *-* | *-* |
|  | AC | chr1p36.13–p32.3, chr9p21.3–p22.2 | - | - |
| 9 | C1 | chr9p23–q22.32, chr18p11.23–q12.3 | chr11 | - |
|  | C2 | *-* | chr5p15.33–q35.3, chr9p24.3 | chr12 |
|  | AC | - | chr11 | chr7p15.1–p11.2 |
| 10 | C1 | chr9p24.3–21.3, chr10, chr18 | - | chr16p13.3, chr17p13.3–12 |
|  | C2 | N/A | N/A | N/A |
|  | AC | chr7q32.1–36.3, chr9p24.3–23, chr10, chr12q15–24.33, chr18q12.1–23 | - | chr1p11.1–q25.2, chr5q13.31–35.3, chr17p13.1–13.1 |
| 16 | C1 | chr9p24.3–p21.3 | - | - |
|  | C2 | chr1p36.33–p36.11, chr1q42.13–q44,  chr9p24.3–p21.3, chr12q21.2–q24.33 | - | - |
|  | AC | N/A | N/A | N/A |
| 17 | C1 | - | chr12, chr14, chr17 | - |
|  | C2 | - | chr14, chr17 | - |
|  | AC | - | chr12, chr14, chr17 | - |
| 18 | C1 | N/A | N/A | N/A |
|  | C2 | N/A | N/A | N/A |
|  | AC | chr9, chr19 | - | - |
| 22 | C1 | chr1p, chr3p–q12.2, chr4, chr6q, chr11q13.5–q21, chr12q23.1–q24.33, chr14q22.3–q32.33,  chr16p13.3, chr22 | - | chr1q, chr13 |
|  | C2 | chr1p, chr3p–q12.2, chr6q, chr16p13.3, chr22 | - | chr1q, chr13 |
|  | AC | chr1p, chr3p–q12.2, chr4, chr6q, chr7q35–q36.3, chr11q14.2–q22.3, chr12q21.33–q24.33, chr14q22.3–q32.33,  chr16p13.3, chr22 | - | chr1q, chr13, chr20, chr21q22.2–q22.3 |
| 23 | C1–MCN | N/A | N/A | N/A |
|  | C2–MCN | N/A | N/A | N/A |
|  | C1–IPMN | - | chr17q | chr18, chr20 |
|  | C2–IPMN | - | chr17q | chr1q21.3–q41, chr20 |
|  | AC | - | chr17q | - |

**Abbreviations:** CNVs, copy number variations; LOH, loss of heterozygosity; N/A, not accessible; MCN, mucinous cystic neoplasm; IPMN, intraductal papillary mucinous neoplasm.

**Table S3**. Differential gene expression analysis (number of overexpressed genes) presented by the different tumor components of SMC

| **SMCs (49)** | **SMC-ACs (8)** |
| --- | --- |
| *ADAMTS1*, *ADAMTSL2*, *ADH1B*, *ALDH1A1*, *C3*, *C7*, *CCDC80*, *CCN2*, *CHRDL1*, *COL14A1*, *CRISPLD2*, *CSRNP1*, *DPT*, *EBF1*, *ELN*, *ERO1B*, *FBLN5*, *FCGBP*, *FOS*, *GPX3*, *HEYL*, *HSPB6*, *IGFBP2*, *INMT*, *INS*, *ITGBL1*, *KANK2*, *LDLRAD4*, *METTL7A*, *MFAP4*, *NFASC*, *NFIX*, *NUDT16*, *PDGFRA*, *PKD1P1*, *PRELP*, *PREX2*, *PTPRS*, *QSER1*, *SLC30A8*, *SMOC2*, *SOD3*, *SORBS2*, *SP4*, *SST*, *SVEP1*, *THSD4*, *ZBTB16*, *ZFP36* | *CXCL5, FRMD6, HSPA1B, HSPH1, LOXL2, MMP11, NEB, SYCP2* |
| **SMC-C1 (19)** | **SMC-ACs (12)** |
| *AC138932.1, ALDH1A1, C7, CCN2, EBF1, ELN, FBLN5, FCGBP, FOS, ITGBL1, KANK2, MFAP4, PTPRS, SLC30A8, SOD3, SST, SVEP1, TERC, ZBTB16* | *AP003100.2, DNAH9, FANCI, GOLGA6L1, LINC01607, LINC01727, MYLK3, OR14J1, OR2AT4, OR2M4, PLCXD2, PRTG* |
| **SMC-C2 (20)** | **SMC-AC (7)** |
| *C7, CCN2, CFTR, ELN, FCGBP, FBLN5, GPC3, GPX3, IGFBP2, LDLRAD4, MFAP4, PRELP, REG1A, REG3A, SOD3, SORBS2, SP4, SST, SVEP1, ZBTB16* | *COL5A3, CXCL5, EREG, HSPA1B, HSPH1, SNORD91B, SYCP2* |
| **SMC-C1 (0)** | **SMC-C2 (3)** |
|  | *MUC4, OR52I2, PRTG* |

**Abbreviations:** SMC, simple mucinous cyst; C1, area with low-grade dysplasia; C2, area with high-grade dysplasia; AC, area with the associated adenocarcinoma.

**Table S4**. Summarizing table of a cohort of mucinous cystic neoplasms without an associated invasive carcinoma

| **ID** | **Sex** | **Age** | **Site** | **Dimension (cm)** | **Dysplasia** | **Follow-up data** |
| --- | --- | --- | --- | --- | --- | --- |
| 1 | M | 64 | Body | 10 | LGD | AFD (>120) |
| 2 | F | 43 | Tail | 6 | LGD | AFD (>120) |
| 3 | F | 69 | Tail | 5 | LGD | AFD (>120) |
| 4 | F | 33 | Tail | 7.5 | LGD | AFD (>120) |
| 5 | F | 27 | Tail | 3.5 | LGD | AFD (>120) |
| 6 | F | 35 | Tail | 7 | LGD | AFD (>120) |
| 7 | F | 40 | Tail | 8 | LGD | AFD (>120) |
| 8 | F | 33 | Body | 2 | LGD | AFD (>120) |
| 9 | F | 31 | Body | 8.5 | LGD | AFD (>120) |
| 10 | F | 66 | Tail | 4 | LGD | AFD (>120) |
| 11 | F | 51 | Body | 3 | LGD | AFD (>120) |
| 12 | F | 58 | Tail | 3.5 | LGD | AFD (>120) |
| 13 | F | 45 | Body | 12 | LGD | AFD (>120) |
| 14 | F | 56 | Body | 2.5 | LGD | AFD (>120) |
| 15 | F | 32 | Tail | 5.5 | LGD | AFD (>120) |
| 16 | F | 36 | Body | 5 | LGD | AFD (>120) |
| 17 | F | 67 | Body | 5 | HGD | AFD (>120) |
| 18 | F | 49 | Tail | 2 | LGD | AFD (>120) |
| 19 | F | 38 | Body | 3.7 | LGD | AFD (>120) |
| 20 | F | 46 | Body | 10.5 | LGD | AFD (>120) |
| 21 | F | 36 | Body | 4 | LGD | AFD (>120) |
| 22 | F | 47 | Tail | 5.5 | LGD | AFD (>120) |
| 23 | F | 33 | Body | 3 | LGD | AFD (>120) |
| 24 | F | 26 | Body | 4.5 | LGD | AFD (>120) |
| 25 | F | 36 | Tail | 7 | LGD | AFD (>120) |
| 26 | F | 72 | Body | 4 | LGD | DOC |
| 27 | F | 70 | Tail | 7 | HGD | AFD (>60) |
| 28 | F | 37 | Tail | 9 | LGD | AFD (>60) |
| 29 | F | 47 | Tail | 7 | LGD | AFD (>60) |
| 30 | F | 46 | Body | 2 | LGD | AFD (>60) |
| 31 | F | 67 | Body | 2 | LGD | AFD (>60) |
| 32 | F | 59 | Head | 2.4 | LGD | AFD (>60) |
| 33 | F | 31 | Body | 5 | LGD | AFD (>60) |
| 34 | F | 28 | Tail | 7 | LGD | AFD (>60) |
| 35 | F | 64 | Tail | 12 | HGD | AFD (>60) |
| 36 | F | 54 | Tail | 5.5 | LGD | AFD (>60) |
| 37 | F | 75 | Body | 2 | LGD | AFD (>60) |
| 38 | F | 48 | Head | 2 | LGD | AFD (>60) |
| 39 | F | 48 | Tail | 5 | LGD | AFD (>60) |
| 40 | F | 33 | Body | 4 | LGD | AFD (>60) |
| 41 | F | 39 | Body | 3 | LGD | AFD (>60) |
| 42 | F | 44 | Tail | 13 | LGD | AFD (>60) |
| 43 | F | 49 | Tail | 3.5 | LGD | AFD (>60) |
| 44 | F | 59 | Body | 2.7 | LGD | AFD (>60) |
| 45 | M | 71 | Head | 4 | LGD | AFD (>60) |
| 46 | F | 34 | Tail | 11 | LGD | AFD (>60) |
| 47 | F | 45 | Tail | 6 | LGD | AFD (>60) |
| 48 | F | 52 | Tail | 3.5 | LGD | AFD (>60) |
| 49 | F | 22 | Head | 9 | LGD | AFD (>60) |
| 50 | F | 65 | Tail | 5 | LGD | AFD (>60) |
| 51 | F | 57 | Tail | 8.5 | HGD | AFD (>60) |
| 52 | F | 52 | Tail | 12 | LGD | AFD (>60) |
| 53 | F | 54 | Body | 3.5 | LGD | AFD (>60) |
| 54 | F | 69 | Head | 2 | LGD | DOC |
| 55 | F | 64 | Body | 6.5 | HGD | AFD (>60) |
| 56 | F | 48 | Tail | 2.5 | LGD | AFD (>60) |
| 57 | F | 36 | Tail | 2.7 | LGD | AFD (>60) |
| 58 | F | 45 | Tail | 5 | LGD | AFD (>60) |
| 59 | F | 52 | Body | 4 | LGD | AFD (>60) |
| 60 | F | 58 | Tail | 8 | HGD | AFD (>60) |
| 61 | F | 56 | Body | 4 | LGD | AFD (>60) |
| 62 | F | 46 | Body | 4 | LGD | AFD (>60) |
| 63 | F | 44 | Body | 6 | LGD | AFD (>60) |
| 64 | F | 36 | Tail | 3.8 | LGD | AFD (>60) |
| 65 | F | 65 | Tail | 4 | LGD | AFD (>60) |
| 66 | F | 44 | Tail | 4 | LGD | AFD (>60) |
| 67 | F | 42 | Tail | 5.7 | LGD | AFD (>60) |
| 68 | F | 39 | Tail | 5.2 | LGD | AFD (>60) |
| 69 | F | 46 | Tail | 4 | LGD | AFD (>60) |
| 70 | F | 51 | Tail | 4.5 | LGD | AFD (>60) |
| 71 | F | 34 | Body | 2.5 | LGD | AFD (>60) |
| 72 | F | 42 | Body | 3 | LGD | AFD (>60) |
| 73 | F | 28 | Tail | 9.5 | LGD | AFD (>60) |
| 74 | F | 67 | Tail | 6 | HGD | AFD (>60) |
| 75 | F | 29 | Body | 4 | LGD | AFD (>60) |
| 76 | F | 62 | Tail | 2.5 | LGD | AFD (>60) |
| 77 | F | 48 | Head | 5.5 | LGD | AFD (>60) |
| 78 | F | 48 | Tail | 3 | LGD | AFD (>60) |
| 79 | F | 47 | Body | 6 | LGD | DOC |
| 80 | F | 55 | Body | 5 | LGD | AFD (>60) |
| 81 | F | 51 | Tail | 4.5 | LGD | AFD (>60) |
| 82 | F | 40 | Tail | 9 | LGD | AFD (>60) |
| 83 | M | 28 | Tail | 4.5 | LGD | AFD (>60) |
| 84 | F | 39 | Body | 4.2 | LGD | AFD (>60) |
| 85 | M | 45 | Tail | 8.5 | LGD | AFD (>60) |

**Abbreviations:** LGD, low-grade dysplasia; HGD, high-grade dysplasia; AFD, alive free of disease; DOC, death from other causes.

**Table S5**. Immunohistochemical scores of mucins in the current case series

| **ID case** | **MUC1** | | **MUC2** | | **MUC4** | | **MUC5AC** | | **MUC6** | |
| --- | --- | --- | --- | --- | --- | --- | --- | --- | --- | --- |
|  | **LGD** | **HGD** | **LGD** | **HGD** | **LGD** | **HGD** | **LGD** | **HGD** | **LGD** | **HGD** |
| 1 | 0 | 0 | 0 | 0 | 9 | 6 | 6 | 6 | 6 | 6 |
| 2 | 6 | 6 | 0 | 0 | 6 | 6 | 9 | 9 | 9 | 9 |
| 3 | 6 | 3 | 3 | 6 | 6 | 6 | 3 | 3 | 0 | 0 |
| 4 | 6 | 6 | 0 | 0 | 6 | 6 | 6 | 6 | 3 | 3 |
| 5 | 6 | 6 | 0 | 3 | 3 | 0 | 6 | 6 | 3 | 3 |
| 6 | 6 | 6 | 0 | 0 | 0 | 0 | 6 | 6 | 0 | 0 |
| 7 | 3 | 3 | 0 | 0 | 6 | 6 | 3 | 3 | 3 | 3 |
| 8 | 6 | 6 | 0 | 0 | 6 | 3 | 6 | 6 | 6 | 6 |
| 9 | 3 | 3 | 0 | 0 | 6 | 6 | 6 | 6 | 3 | 3 |
| 10 | 0 | 3 | 0 | 0 | 3 | 3 | 0 | 6 | 0 | 0 |
| 11 | 3 | 3 | 0 | 0 | 3 | 6 | 6 | 6 | 3 | 3 |
| 12 | 0 | 0 | 0 | 0 | 6 | 9 | 3 | 3 | 0 | 0 |
| 13 | 0 | 0 | 0 | 0 | 9 | 6 | 9 | 9 | 9 | 9 |
| 14 | 0 | 0 | 9 | 9 | 6 | 6 | 9 | 9 | 0 | 0 |
| 15 | 0 | 3 | 0 | 0 | 3 | 0 | 0 | 0 | 3 | 0 |
| 16 | 9 | 6 | 0 | 3 | 3 | 6 | 6 | 3 | 0 | 3 |
| 17 | 3 | 3 | 3 | 6 | 0 | 6 | 0 | 3 | 0 | 0 |
| 18 | 0 | 3 | 0 | 0 | 3 | 3 | 0 | 6 | 0 | 0 |
| 19 | 6 | 6 | 0 | 0 | 6 | 6 | 3 | 3 | 3 | 3 |
| 20 | 3 | 3 | 0 | 0 | 0 | 0 | 0 | 0 | 0 | 3 |
| 21 | 0 | 0 | 3 | 0 | 0 | 3 | 3 | 6 | 6 | 6 |
| 22 | 0 | 0 | 0 | 0 | 3 | 0 | 0 | 0 | 3 | 0 |
| % Positive cases (mean value if positive) | MCN 61.1% (5.2)  SMC 50.0% (4.5) | MCN 77.7% (3.3)  SMC 50.0% (4.5) | MCN 16.6% (5.0)  SMC 25.0% (3.0) | MCN 27.7% (5.4)  SMC 0.0% (NA) | MCN 88.8% (5.2)  SMC 50.0% (4.5) | MCN 83.3% (5.6)  SMC 50.0% (4.5) | MCN 77.7% (6.0)  SMC 50.0% (3.0) | MCN 94.4% (5.6)  SMC 50.0% (4.5) | MCN 55.5% (4.8)  SMC 75.0% (4.0) | MCN 55.5% (4.8)  SMC 75.0% (4.0) |

**Abbreviations:** MCN, mucinous cystic neoplasm (cases no. 1–18); SMC, simple mucinous cyst (cases no. 19–22); LGD, low-grade dysplasia (represents the C1 sample); HGD, high-grade dysplasia (represents the C2 sample).
